# COVID-19 vaccine uptake, effectiveness, and waning in 82,959 health care workers: A national prospective cohort study in Wales

**DOI:** 10.1016/j.vaccine.2021.11.061

**Published:** 2022-02-16

**Authors:** Stuart Bedston, Ashley Akbari, Christopher I. Jarvis, Emily Lowthian, Fatemeh Torabi, Laura North, Jane Lyons, Malorie Perry, Lucy J. Griffiths, Rhiannon K. Owen, Jillian Beggs, Antony Chuter, Declan T. Bradley, Simon de Lusignan, Richard Fry, F.D. Richard Hobbs, Joe Hollinghurst, Srinivasa Vittal Katikireddi, Siobhán Murphy, Dermot O'Reily, Chris Robertson, Ting Shi, Ruby S.M. Tsang, Aziz Sheikh, Ronan A. Lyons

**Affiliations:** a Population Data Science, Swansea University Medical School, Swansea University, UK; b London School of Hygiene and Tropical Medicine, UK; c Vaccine Preventable Disease Programme, Public Health Wales, Cardiff, UK; d Patient & Public Involvement (PPI) BREATHE – The Health Data Research Hub for Respiratory Health, UK; e Centre for Public Health, Queen’s University Belfast, UK; f Nuffield Department of Primary Care Health Sciences, University of Oxford, UK; g MRC/CSO Social & Public Health Sciences Unit, University of Glasgow, UK; h Department of Mathematics and Statistics, Strathclyde University, Glasgow and Public Health Scotland, UK; i Usher Institute, University of Edinburgh, UK; j Usher Institute and HDR UK BREATHE Hub, University of Edinburgh, UK

**Keywords:** COVID-19, Health care workers, Vaccines, Pandemic

## Abstract

**Background:**

While population estimates suggest high vaccine effectiveness against SARS-CoV-2 infection, the protection for health care workers, who are at higher risk of SARS-CoV-2 exposure, is less understood.

**Methods:**

We conducted a national cohort study of health care workers in Wales (UK) from 7 December 2020 to 30 September 2021. We examined uptake of any COVID-19 vaccine, and the effectiveness of BNT162b2 mRNA (Pfizer-BioNTech) against polymerase chain reaction (PCR) confirmed SARS-CoV-2 infection. We used linked and routinely collected national-scale data within the SAIL Databank. Data were available on 82,959 health care workers in Wales, with exposure extending to 26 weeks after second doses.

**Results:**

Overall vaccine uptake was high (90%), with most health care workers receiving the BNT162b2 vaccine (79%). Vaccine uptake differed by age, staff role, socioeconomic status; those aged 50–59 and 60+ years old were 1.6 times more likely to get vaccinated than those aged 16–29. Medical and dental staff, and Allied Health Practitioners were 1.5 and 1.1 times more likely to get vaccinated, compared to nursing and midwifery staff. The effectiveness of the BNT162b2 vaccine was found to be strong and consistent across the characteristics considered; 52% three to six weeks after first dose, 86% from two weeks after second dose, though this declined to 53% from 22 weeks after the second dose.

**Conclusions:**

With some variation in rate of uptake, those who were vaccinated had a reduced risk of PCR-confirmed SARS-CoV-2 infection, compared to those unvaccinated. Second dose has provided stronger protection for longer than first dose but our study is consistent with waning from seven weeks onwards.

## Highlights

• 90% vaccine uptake by 30 Sept 2021, but uptake in more deprived areas was lower.
• BNT162b2 was 85% effective after 2 weeks from second dose and 52% from 22 weeks.
• Equitable effectiveness of BNT162b2 after second dose.

## 1 Introduction

The devastation, disruption, and daunting scale of the COVID-19 pandemic has perhaps been matched by the fastest development of vaccines in history [1], [2]. Vaccine efficacy for COVID-19 vaccines has been measured in the sanitised environment of clinical trials; however, determining the real-world impact of these vaccines requires consideration of how many people will take them (*uptake*), the reduction in serious outcomes (*effectiveness*), and how long they provide protection for (*waning*).

Health care workers (HCWs) are at increased risk of exposure to severe acute respiratory syndrome coronavirus 2 (SARS-CoV-2; the cause of COVID-19) when compared to the general population [3]. Thus, studying this cohort provides insight for how effective the vaccines are in an environment where risk of exposure is higher than in the general population, but with potentially stronger adherence to individual protective behaviours that aim to reduce transmission. In Wales, HCWs were required to take a polymerase chain reaction (PCR) test if they showed symptoms or were identified as being in contact with someone who had tested positive for COVID-19. Symptoms included: high temperature, continuous cough, and a loss or change to sense of taste or smell [4].

The vaccination programme in Wales officially started on 8 December 2020, initially with only the BNT162b2 mRNA (Pfizer-BioNTech) vaccine being available, from 4 January 2021 ChAdOx1 adenoviral (Oxford-AstraZeneca) became available, and then mRNA-1273 (Moderna) from 7 April 2021 [5]. Each of these vaccines were on a two-dose schedule, with the recommendation that there be 8–12 weeks between doses [6]. Priority was initially given to health care workers and those at highest risk of COVID-19 hospitalisation and death [7]. From 20 September 2021, booster vaccinations were offered to those of priority which included health care workers, no earlier than six months after their second dose [8].

We constructed a national cohort of HCWs to describe uptake of COVID-19 vaccines, effectiveness, and waning of the BNT162b2 vaccine (VE) against PCR-confirmed infection following first and second doses up to 26 weeks post-vaccination.

## 2 Materials and methods

We constructed a prospective, national-scale, observational cohort of HCWs in Wales. We used individual-level, linked, anonymised electronic health record and administrative data sources available within the Secure Anonymised Information Linkage (SAIL) Databank [9]. These data sources included demographics, address history, laboratory testing, vaccination, General Practitioner (GP) diagnoses, prescribed and dispensed medications, hospital admissions, and death (Supplementary Material S1) [10]. We conducted two analyses on this cohort. Firstly, we analysed uptake of COVID-19 vaccines for the period 7 December 2020 (start of programme) to 11 June 2021. Secondly, we analysed vaccine effectiveness against SARS-CoV-2 infection confirmed by positive PCR test. For both stages, we present unadjusted and adjusted results based on Cox Proportional Hazard models. We also have followed the STROBE checklist in our reporting.

### 2.1 Characteristics and confounders

We chose HCW staff role, age, sex, ethnicity, and socioeconomic status (SES) at baseline as relevant characteristics. The following were considered as confounders: urban/rural classification of the HCW home address, number of QCovid co-morbidities at baseline, number of PCR tests taken before 7 December 2020, health service utilisation (number of hospital admissions, GP attendances and prescriptions, separately, over the last five years) and residing health board (geographical NHS administrative area) [11], [12]. Staff roles were categorised into nine groups: nursing and midwifery, clinical services, administrative, estates and ancillary (e.g. porter, security, housekeeping, catering), medical and dental, Allied Health Professionals, technical staff, healthcare scientists, and students. SES quintiles were defined by linking the 2019 Welsh Index of Multiple Deprivation (WIMD) to the 2011 Lower-layer Super Output Area (LSOA) of each HCW's residence [13]. Due to potential disclosure risk, we aggregated available ethnicity records into groups ‘White’ and 'minority ethnic’ (Asian, Black, Mixed and Other) [14]. Co-morbidities are listed Table S2 in the Supplementary Material. From these, body mass index (BMI) was the only measure with missing data (47% missing based on last five years). Due to computational efficiency, we performed a single imputation of BMI [15], for inclusion with other indicators to produce a co-morbidity score with intervals: (low) 0, 1, 2, 3 or more (high).

### 2.2 Analysis of COVID-19 vaccine uptake

The primary outcome was time until first dose of any available COVID-19 vaccination. We used Cox proportional hazard models to analyse the length of time from 7 December 2020 to the date of administration, reporting unadjusted and adjusted hazard ratios (aHR) with 95% confidence intervals (95 %CI) based on robust standard errors. We censored observations if individuals became infected, were no longer employed as a HCW, moved out of Wales, or died before vaccination. aHRs were estimated given other characteristics and confounders, stratifying by previous number of PCR tests. We included separate step-time-varying coefficients for both administrative staff and the minority ethnic group as we observed non-proportionality against the baseline for these groups.

### 2.3 Analysis of BNT162b2 vaccine effectiveness

The primary outcome of interest was time until infection determined by a positive PCR test from 7 December 2020 onwards. We defined effectiveness as being the point at which the BNT162b2 vaccine provides a 50% reduction in the relative risk of infection compared to unvaccinated [16], [17].

Secondary outcomes of interest were whether COVID-19 infection was associated with hospitalisation or death. We defined an individual as having a COVID-19 related hospitalisation if they had an admission from the day before the positive test up to 28 days after. Death was recorded from several data sources (Table S1 in Supplementary Material).

We focused our analysis of VE on the relative risk of a positive PCR test as the number of hospitalisations and death was low. We analysed the effectiveness of first and second dose jointly, relative to being unvaccinated. We used Cox proportional hazard models, censoring when someone received a vaccine different from BNT162b2, received the booster, died (unrelated to COVID-19), were no longer employed as an HCW, or moved out of Wales. Overall VE was modelled using two penalised splines given continuous number days vaccinated with one and two doses. Based on these results, for characteristic-specific VE estimates were subsequently modelled separately as an interaction between the characteristic of interest and discrete intervals of days vaccinated. For which we present the aHR for first at 3–6 weeks, second dose at 2–5 weeks and 22–25 weeks, with 95% CIs based on robust standard errors. Unadjusted and adjusted estimates for all intervals are reported in Table S5 the Supplementary Material.

### 2.4 Sensitivity analyses

**Fig. 1:**
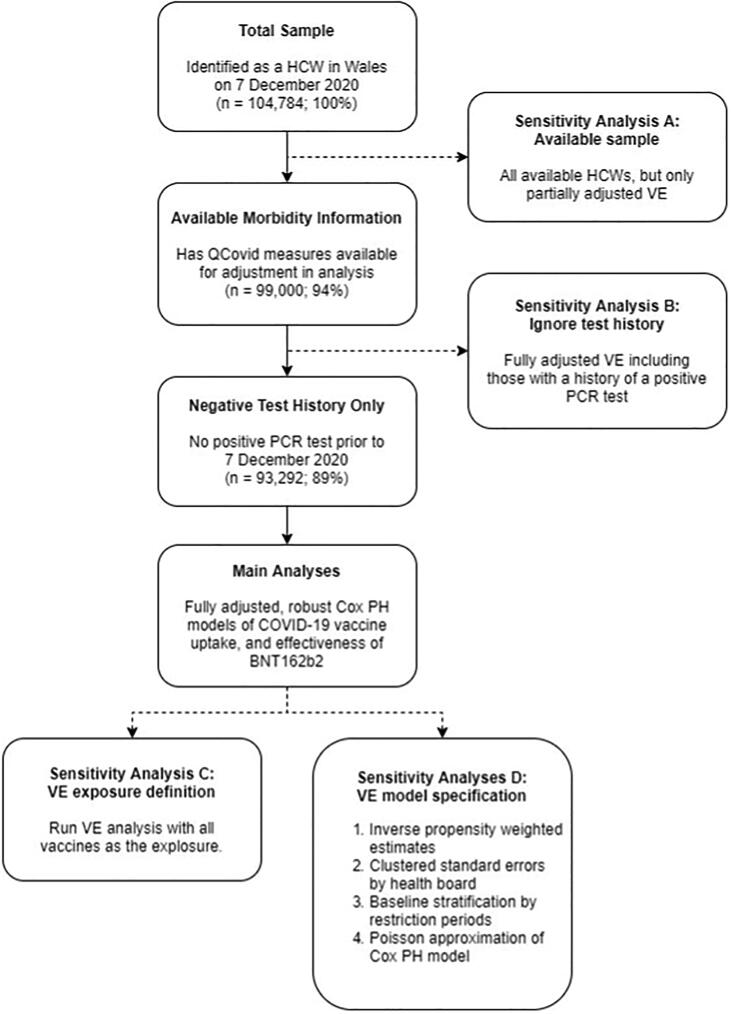
Flowchart of sample selection, main analyses and sensitivity analyses A-D.

We conducted seven specific sensitivity analyses related to overall BNT162b2 VE (A-D, Fig. 1) to consider the assumptions and choices made for sample selection (A), test history (B), exposure definition (C), and model specification (D). For sample selection (A), we repeated the main analysis on all available HCWs, though we used a simpler model as co-morbidity measures were unavailable in 20% of the population. We considered ignoring tests prior to the study window (B), repeating the analysis for HCWs with available co-morbidity measures irrespective of whether they had previously tested positive for COVID-19. For exposure definition (C), we only considered being vaccinated with any COVID-19 vaccine as the exposure. For model specifications (D), we fitted four alternative models (D1-D4). For D1, the sample was inverse probability weighted based on uptake of first dose by 25 January 2021, addressing potential bias in the estimates of the non-random uptake of the vaccine. For D2, standard errors were clustered by health board, this addresses potential dependence between HCWs from the same health board. Additionally, for D3, we stratified the baseline by restriction periods to determine if these dramatic changes in background risk were affecting proportionality. Finally, for D4 we approximated the Cox proportional hazards model with a Poisson model with rate of infection being assessed across weekly intervals.

## 3 Results

There were 104,784 potentially eligible HCWs employed by NHS Wales on 7 December 2020. Of these, 88,225 (Δ = -16,559) had available records on co-morbidities related to COVID-19. Excluding those (Δ = -5,266) who had tested positive for COVID-19 prior to 7 December 2020, the cohort compromised of 82,959 HCWs (Fig. 1, Table 1) who yielded a total of 61,164 person-years of follow-up, with an average follow-up period of 269 days. Over the study window, only 4.6% of HCWs left their HCW role or moved out of Wales.

**Table 1:** Descriptive summaries of health care workers (n = 82,959).

		n	Col. %
COVID-19 related outcome	Death caused by COVID-19	<10	
	Hospitalisation within 28 days	<250	
	Positive PCR test only	9,081	10.9%
	None	73,619	88.7%
First vaccine dose	Unvaccinated	8,569	10.3%
	ChAdOx1	8,799	10.6%
	mRNA-1273	277	0.3%
	BNT162b2	65,314	78.7%
Last known vaccination status	Unvaccinated	8,569	10.3%
	ChAdOx1 Dose 1	523	0.6%
	ChAdOx1 Dose 2	8,078	9.7%
	mRNA-1273 Dose 1	74	0.1%
	mRNA-1273 Dose 2	203	0.2%
	BNT162b2 Dose 1	2,840	3.4%
	BNT162b2 Dose 2	43,175	52.0%
	Booster	19,497	23.5%
Patient facing	Yes	50,213	60.5%
	Undetermined	20,097	24.2%
	No	12,649	15.2%
Staff group	Clinical services	21,024	25.3%
	Nursing and midwifery	21,122	25.5%
	Admin	17,091	20.6%
	Estates and ancillary	7,634	9.2%
	Medical and dental	6,305	7.6%
	Allied Health Professionals	5,165	6.2%
	Technical	2,760	3.3%
	Healthcare Scientists	1,729	2.1%
	Students	129	0.2%
Prior PCR test history	0	59,491	71.7%
	1	16,569	20.0%
	2	4,339	5.2%
	3+	2,560	3.1%
Sex	Male	18,551	22.4%
	Female	64,408	77.6%
Age	16–29	13,346	16.1%
	30–39	19,159	23.1%
	40–49	19,229	23.2%
	50–59	22,509	27.1%
	60+	8,716	10.5%
Ethnicity	White	75,621	91.2%
	Minority ethnic	6,524	7.9%
	(Missing)	814	1.0%
Urban/rural classification	Urban City And Town	57,789	69.7%
	Rural Town And Fringe	12,302	14.8%
	Rural Village And Dispersed	8,415	10.1%
	(Missing)	4,453	5.4%
SES quintile	1st (Most deprived)	12,468	15.0%
	2nd	15,894	19.2%
	3rd	16,086	19.4%
	4th	17,333	20.9%
	5th (Least deprived)	21,178	25.5%
Hospital admissions (previous 5 years)	0	48,159	58.1%
	1	15,842	19.1%
	2+	18,958	22.9%
GP attendances (previous 5 years)	0	131	0.2%
	1*–*19	18,224	22.0%
	20*–*39	24,958	30.1%
	40*–*59	17,489	21.1%
	60+	22,157	26.7%
Prescriptions (previous 5 years)	0	5,317	6.4%
	1*–*9	22,623	27.3%
	10*–*49	33,144	40.0%
	50+	21,875	26.4%
Health board	Betsi Cadwaladr University Health Board	16,511	19.9%
	Cardiff and Vale University Health Board	16,021	19.3%
	Aneurin Bevan University Health Board	12,657	15.3%
	Cwm Taf Morgannwg University Health Board	10,157	12.2%
	Swansea Bay University Health Board	16,924	20.4%
	Hywel Dda University Health Board	9,770	11.8%
	Powys Teaching Health Board	919	1.1%
QCOVID co-morbidity score	0	35,527	42.8%
	1	30,871	37.2%
	2	12,192	14.7%
	3+	4,369	5.3%

The most common staff groups were nursing and midwifery (n = 21,122, 25.5%; Table 1) and clinical services (25.3%). Most HCWs were female (77.6%) and aged between 50 and 59 years old (27.1%). The majority of HCWs were White (91.2%), resided in an area classified as urban city and town (69.7%). A quarter were living in the least deprived quintile (25.5%), whereas 15.0% were in the most deprived quintile. Most HCWs had zero QCovid co-morbidities (42.8%), a similar number had only one (37.2%), fewer had two (14.7%), and a low proportion had three or more (5.3%). Of the 82,959 HCWs, 9,081 (10.9%) had a positive PCR test, less than 250 were hospitalised and less than 10 died due to COVID-19 during follow-up.

### 3.1 COVID-19 vaccine uptake

**Fig. 2:**
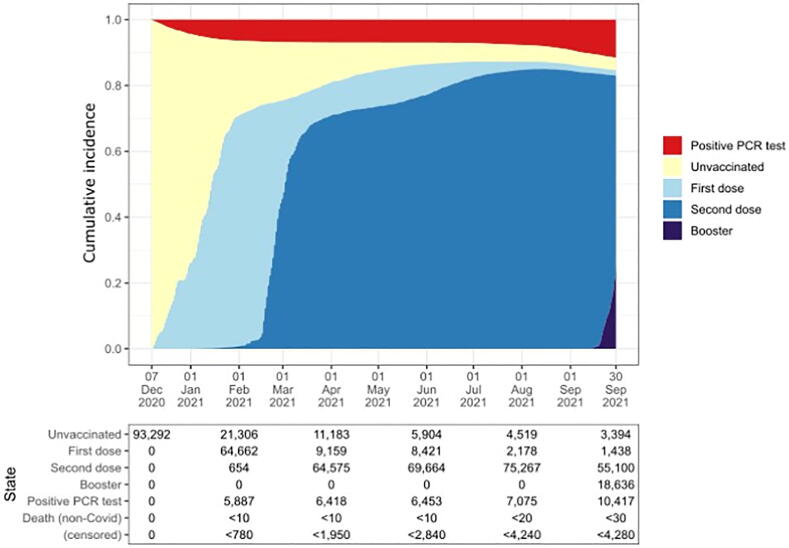
Cumulative incidence of vaccination status and PCR-confirmed COVID-19 infection for health care workers (n = 93,292), in Wales, from 7 December 2020 to 30 September 2021.

Over the entire follow-up period, 89.7% (n = 74,489) had at least one dose, with 85.5% (n = 70,953) having had two doses of a vaccine (Table 1). Most HCWs received BNT162b2 (n = 65,314, 78.7%), compared to ChAdOx1 (n = 8,799, 10.6%) and mRNA-1273 (n = 277, 0.3%). From the start, there was substantial growth in the uptake of the vaccine among HCWs, increasing steadily until February 2021, and more slowly thereafter (Fig. 2). Uptake of second dose rose rapidly from mid-February, with 57,299 of HCWs having been administered with their second dose as early as April 2021. This uptake of the vaccine happened against a background of increasing number of confirmed cases in the general population, which plateaued from February 2021, but then saw a new rate of increase from August 2021 [18].

From our analysis, those aged 50*–*59 were 1.60 times more likely to receive the vaccine compared to those aged 16*–*29 (aHR 1.60, 95% CI 1.56*–*1.64; Table 2). Medical and dental staff were nearly 1.46 times more likely than nursing and midwife (aHR 1.46, 95% CI 1.41*–*1.52), whilst admin staff were 53% less likely in the first 99 days of the vaccination programme (aHR 0.47, 95% CI 0.46*–*0.48), but twice as likely from 100 days onwards (aHR 1.88, 95% CI 1.79*–*1.97). The uptake of vaccine was lower among those from minority ethnic groups compared to the White group (aHR 0.93, 95% CI 0.90*–*0.96); this difference increased from 51 days onwards (aHR 0.72, 95% CI 0.68*–*0.76). We also found that those living in the most affluent areas had a higher relative rate of vaccine uptake compared to those living in the most deprived areas (aHR 1.12, 95% CI 1.09*–*1.15).

**Table 2:** Hazard ratios (95% confidence interval) of COVID-19 vaccine uptake by health care work characteristics, from 7 December 2020 to 30 September 2021.

				Unadjusted*		Adjusted**	
Characteristic		Person-years	Vaccinations	HR	95% CI	HR	95% CI
**Staff group**	Nursing and midwifery	2,736	19,125	1.00		1.00	
	Clinical services	3,452	18,210	0.76	(0.74 to 0.77)	0.80	(0.78 to 0.81)
	Admin						
	0–99 days	2,632	11,666	0.49	(0.48 to 0.51)	0.47	(0.46 to 0.48)
	100 + days	737	3,787	1.88	(1.79 to 1.97)	1.88	(1.79 to 1.97)
	Estates and ancillary	1,215	6,701	0.76	(0.74 to 0.78)	0.70	(0.69 to 0.73)
	Medical and dental	623	5,877	1.38	(1.33 to 1.43)	1.46	(1.41 to 1.52)
	Allied Health Professionals	635	4,747	1.07	(1.04 to 1.11)	1.12	(1.08 to 1.16)
	Technical	336	2,557	1.08	(1.03 to 1.13)	1.11	(1.06 to 1.16)
	Healthcare scientists	231	1,598	0.94	(0.90 to 0.99)	0.95	(0.90 to 1.00)
	Students	22	108	0.71	(0.59 to 0.84)	0.84	(0.70 to 1.00)
**Sex**	Female	9,956	57,609	1.00		1.00	
	Male	2,666	16,767	1.09	(1.07 to 1.11)	1.04	(1.02 to 1.06)
**Age**	16–29	2,645	11,242	1.00		1.00	
	30–39	3,525	16,727	1.11	(1.08 to 1.14)	1.08	(1.05 to 1.10)
	40–49	2,713	17,432	1.44	(1.41 to 1.47)	1.38	(1.35 to 1.42)
	50–59	2,731	20,846	1.65	(1.61 to 1.69)	1.60	(1.56 to 1.64)
	60+	1,007	8,129	1.69	(1.64 to 1.73)	1.65	(1.60 to 1.70)
**Ethnicity**	White	11,488	67,919	1.00		1.00	
	Minority ethnic						
	0–50 days	541	4,540	1.12	(1.08 to 1.16)	0.93	(0.90 to 0.96)
	51 + Days	452	1,202	0.79	(0.74 to 0.83)	0.72	(0.68 to 0.76)
	(Missing)	141	715	0.88	(0.82 to 0.95)	0.75	(0.69 to 0.81)
**SES quintile**	1st (Most deprived)	2,131	10,840	1.00		1.00	
	2nd	2,486	14,055	1.09	(1.07 to 1.12)	1.05	(1.03 to 1.08)
	3rd	2,549	14,371	1.09	(1.06 to 1.12)	1.03	(1.00 to 1.05)
	4th	2,533	15,692	1.18	(1.15 to 1.21)	1.09	(1.06 to 1.12)
	5th (Least deprived)	2,922	19,418	1.27	(1.24 to 1.30)	1.12	(1.09 to 1.15)

* Main effect only, with stratification by 'prior PCR test history'.

** Main effects included staff group, sex, age, ethnicity, SES, urban/rural classification, QCovid co-morbidity score, previous number of hospitlisations, GP attendances and prescriptions over the last five years, with stratificaiton by 'prior PCR test history'.

### 3.2 BNT162b2 vaccine effectiveness

#### 3.2.1 First dose

**Fig. 3:**
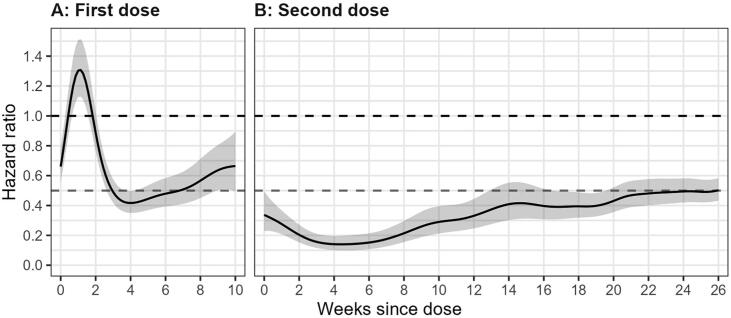
Adjusted hazard ratios (95% confidence interval) for BNT162b2 effectiveness against PCR-confirmed COVID-19 infection in health care workers over time, for (A) first and (B) second dose, relative to unvaccinated. Dashed lines at 1.0 and 0.5.

**Fig. 4:**
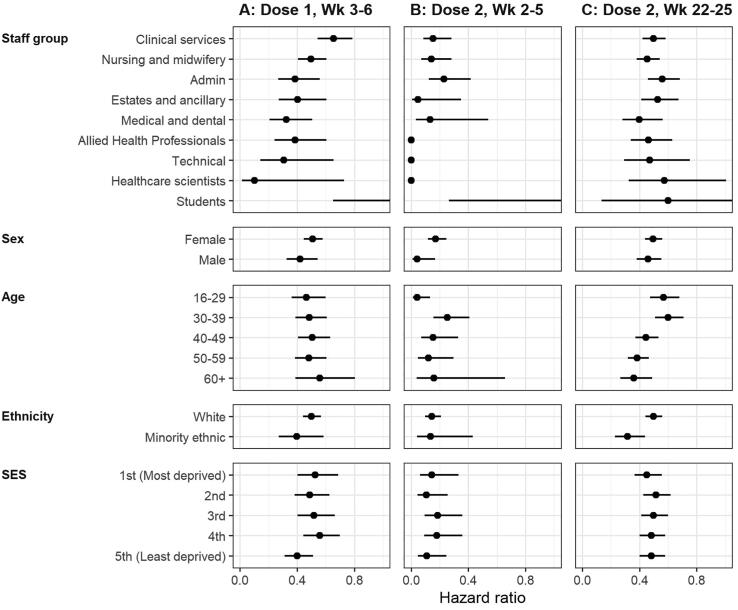
Adjusted hazard ratios (95% confidence interval) for BNT162b2 vaccine effectiveness against PCR-confirmed COVID-19 infection for (A) first dose weeks 3–6, (B) second dose weeks 2–5 and (C) weeks 22–25. All cases were vaccinated between 7 December 2020 and 30 September 2021. Estimates shown are relative to the same characteristic unvaccinated.

We found no decrease in risk of PCR-confirmed COVID-19 infection in the first two weeks following a first dose of BNT162b2, relative to being unvaccinated (Fig. 3A, Table 3). From week three and persisting until week six, we observed a vaccine effectiveness of 52% (aHR 0.48, 95 %CI 0.42*–*0.55). At peak effectiveness (weeks 3 to 6), only slight differences were found amongst staff groups (Fig. 4A); ignoring students due to small numbers, additional clinical services had the lowest vaccine effectiveness (aHR 0.65, 95 %CI 0.54*–*0.79). Little differences were found by sex, age, ethnicity and SES.

**Table 3:** Hazard ratios (95% confidence interval) for BNT162b2 effectiveness against PCR-confirmed COVID-19 infection in health care workers, relative to unvaccinated.

				Unadjusted*		Adjusted**	
Dose	Week	Person-years	Events†	HR	95% CI	HR	95% CI
Unvaccinated		12,748	4,680	1.00		1.00	
First	0–2	4,028	1,080	0.94	(0.88, 1.01)	0.97	(0.90 to 1.04)
	3–6	4,982	310	0.47	(0.41, 0.53)	0.48	(0.42 to 0.55)
	7+	2,123	140	0.60	(0.49, 0.74)	0.61	(0.50 to 0.76)
Second	0–1	2,711	60	0.31	(0.23, 0.42)	0.33	(0.24 to 0.44)
	2–5	5,372	50	0.13	(0.09, 0.20)	0.14	(0.09 to 0.21)
	6–13	10,414	230	0.21	(0.16, 0.28)	0.23	(0.17 to 0.31)
	14–17	4,849	180	0.36	(0.28, 0.46)	0.40	(0.31 to 0.52)
	18–21	4,554	360	0.38	(0.32, 0.45)	0.43	(0.36 to 0.50)
	22–25	4,341	670	0.42	(0.37, 0.48)	0.47	(0.41 to 0.53)
	26+	4,997	1,570	0.48	(0.43, 0.54)	0.55	(0.49 to 0.61)

* Only stratification by categories of prior number of PCR tests.

** Main effects included staff group, sex, age, ethnicity, SES, urban/rural classification, QCovid co-morbidity score, previous number of hospitalisations, GP attendances and prescriptions over the last five years, with stratification by 'prior PCR test history'.

† For disclosure purposes, number of events have been round to nearest 10.

#### 3.2.2 Second dose

We found a substantial reduction in risk of PCR-confirmed COVID-19 infection for vaccinated HCWs relative to unvaccinated (Fig. 3B, Table 3). By the end of the first two weeks, vaccine effectiveness was 67% (aHR 0.33, 95 %CI 0.24*–*0.44). This increased in weeks 2*–*5 to 86% (aHR 0.14, 95 %CI 0.09*–*0.21), and decreased to 77% over weeks 6*–*13. After this, vaccine effectiveness decreased from 60% to 53% between weeks 14*–*25, and from week 26 vaccine effective was 45% (aHR 0.55, 95 %CI 0.49*–*0.61). Additionally, vaccine effectiveness was considerably equitable across characteristics (Fig. 4B and 4C). In weeks 2*–*5 there were little differences across the staff groups (students have a small number of observations), sex, age, ethnicity and deprivation. For weeks 22*–*25, all characteristics appeared to have somewhat equal effectiveness, however those aged 16*–*29 (aHR 0.54, 95 %CI 0.44*–*0.65) and 30*–3*9 (aHR 0.61, 95 %CI 0.52*–*0.72) had lower rates of effectiveness than the older age groups; 40*–*49 (aHR 0.45, 95 %CI 0.37*–*0.54).

### 3.3 Sensitivity analyses

Results from the sensitivity analyses were broadly complementary and reinforced the main findings (Figure S5, Table S4 in Supplementary Material). There was only one exception which was the inverse propensity weighted (IPW) estimates of VE, which showed first dose achieving 50% effectiveness slightly earlier than our main analysis. However, beyond this the IPW estimates where inline with our main analysis.

## 4 Discussion

Our national cohort study has found overall high vaccine uptake in HCWs, and high vaccine effectiveness for BNT162b2. Despite the potential for increased risk of exposure for HCWs, it appears that two doses of the BNT162b2 vaccine was able to provide considerable protection of up to 85% effectiveness against COVID-19 infection. However, we found evidence of waning, with effectiveness being 53% by six months after the second dose. Following this, our study provides further evidence for the Welsh vaccination strategy which currently advises that frontline health and social care workers receive a booster vaccine after 6 months [19]. Given that most previous studies on VE for HCWs have only been able to rely on self-reported data [20], and smaller samples [21], [22], this study contributes uniquely in two ways. First, it provides national population estimates over time of vaccine uptake and effectiveness for HCWs in relation to PCR-test-confirmed infection. Second, it provides estimates of uptake and effectiveness across a range of sociodemographic characteristics, most notably health care occupation, which has not been conducted even in larger cohorts of HCWs [23].

We found variation in vaccine uptake across staff groups and age. This likely reflects that there were other factors beyond viewing HCWs as a whole priority group for vaccination, such as people’s vulnerability profile, age, and social factors. This finding is consistent with the pattern of general health in HCWs and aligns with Hall et al. who also found older HCWs were more likely to be vaccinated [20], [24]. Our analysis also found that estates and ancillary staff were less likely to be vaccinated than nursing and midwifery; although comparisons are limited due to differing health care worker definitions in other studies. Consistent with other studies [20], [25], [26], we observed that vaccine uptake was lower for more deprived groups, this was despite adjusting for multiple factors which have socioeconomic variance, such as staff group. While this effect was small, the notion that more affluent groups are better placed to benefit from universal healthcare is observed [27], likely reflecting differences in access, resources (e.g. time), and distrust, as highlighted in Dickerson et al. [25].

The BNT162b2 vaccine was clearly effective with a strong reduction in risk of infection three weeks after the first vaccine. This is consistent with other research examining HCWs, with estimates of VE ranging from 60 to 75% after at least two weeks from first dose [20], [21], [28], [29], though one study found only 17% VE after 14 days [30]. Likewise, our study is comparable to those focusing on the general, older population [31]. Indeed, our data are strongly suggestive of the additional benefit of the second dose, as in previous studies, with effectiveness around 85% after two weeks from second dose, which again, is consistent with previous studies [20], [22], [28], [29], [30], [31], [32], [33]. We provide one of the largest national studies considering vaccine uptake and effectiveness across different HCW roles. Variability in our findings may reflect differentials in risk profiles, with those working in riskier settings more likely to be exposed to COVID-19 patients following a first dose compared to those unvaccinated. These potential differences should be treated with caution, as after the second dose, variability of effectiveness is minimal with nearly all characteristics achieving 80% VE by week two.

It is plausible that the reduction in positive tests among vaccinated individuals was influenced by other factors, such as non-pharmaceutical interventions (NPIs), which were in place during this period. It is unlikely that the strong effects seen were the impact of restrictions alone, particularly as positive COVID-19 tests have not grown substantially among vaccinated HCW (see Fig. 1). Likewise, the results show a strong dose response relationship of a large magnitude and are consistent with the efficacy measurements established in clinical trials. Furthermore, any impact of NPIs would need to have a differential protective impact for vaccinated versus non-vaccinated, which is unlikely to be the case. Indeed, most plausible differential exposure of vaccinated individuals, such as might be achieved from vaccine passports (not yet implemented in Wales), would likely increase rather than decrease their risk.

Our interpretation of effectiveness is complicated due to limited data available on COVID-19 variants. The Alpha variant was the dominant strain in Wales from December 2020 to May 2021, with the Delta variant rapidly taking over from June 2021 onwards. Whilst vaccinations have been found to be less effective against the Delta variant [34], we surmise that BNT162b2 still provided protection to HCWs. However, if there is a true difference in VE between these variants, future analyses would require this information, otherwise measures of effectiveness will average across variants and not represent a single effect.

Consistent with other research, we found slightly lower levels of uptake in minority ethnic HCWs [20]. Though the number of people from a minority ethnic group make up a small proportion of the sample (7.9%) this is larger than seen in the wider Wales population [35]. It is important to note that the binary measure of ethnicity was only used due to disclosure risks and the ethnic minority group category here represents an average of likely diverse and differing effects, and therefore should be interpreted with caution. Further analyses on larger scale data should be conducted to consider this aspect of vaccine uptake, such as in Hall et al. [20]. We were fortunate to have a national cohort of HCWs across many staff areas, which allowed us to consider the importance of occupational role in vaccine uptake and effectiveness. Our study is strengthened by the longitudinal design and followed the same individuals over time thus reducing some of the aspects of individual variation between comparisons of different time points. Most importantly, due to the range of information available, we were able to provide a holistic picture on uptake and effectiveness.

## 5 Conclusion

Our study provides strong evidence for vaccine uptake and effectiveness for HCW’s in a socioeconomically diverse setting in the UK. Vaccine uptake was high overall, but with some variation; in particular, younger, more deprived, minority ethnic staff and those in non-patient facing roles were least likely to be vaccinated. From this, we recommend that policymakers and practitioners place greater efforts on addressing vaccine hesitancy for HCW’s in more deprived settings given their inherent vulnerability and risk for testing positive. This is particularly important given VE was 86% at around 2 weeks, and remained over 50% effective until 26 weeks (or 6 months). Moreover, effectiveness was equitable across sociodemographic characteristics, which providers further support for addressing the vaccine gap for disadvantaged HCW’s. In short, we find strong evidence to suggest two doses of vaccine provided considerable additional protection to HCWs despite their increased risk of exposure to COVID-19.

## Declaration of Competing Interest

The authors declare the following financial interests/personal relationships which may be considered as potential competing interests: AS is a member of the Scottish Government Chief Medical Officer’s COVID-19 Advisory Group, the New and Emerging Respiratory Virus Threats (NERVTAG) Risk Stratification Subgroup and a member of AstraZeneca’s Thrombotic Thrombocytopenic Task Force; all roles are unremunerated. CR is a member of the Scottish Government Chief Medical Officer’s COVID-19 Advisory Group, the Scientific Pandemic Influenza Group on Modelling and the Medicines & Healthcare products Regulatory Agency’s Vaccine Benefit and Risk Working Group. DTB is employed by the Public Health Agency, Northern Ireland, and the Department of Health, Northern Ireland. MP is employed by the Vaccine Preventable Disease Programme at Public Health Wales and is involved in the COVID-19 vaccine roll out for Wales. SVK is co-chair of the Scottish Government’s Expert Reference Group on COVID-19 and ethnicity, is a member of the Scientific Advisory Group on Emergencies (SAGE) subgroup on ethnicity and acknowledges funding from a NRS Senior Clinical Fellowship, MRC and CSO. RF is a member of is a member of the Scientific Advisory Group on Emergencies (SAGE) Social Care Working Group (SCWG). RO is a member of the National Institute for Health and Care Excellence (NICE) Technology Appraisal Committee (TAC) and has provided methodological advice outside of the submitted work to Cogentia Healthcare Consulting Ltd, F. Hoffmann-La Roche Ltd, the National Institute for Health and Care Excellence (NICE) Decision Support Unit, the University of Bristol, and the Association of the British Pharmaceutical Industry (ABPI). RAL is a member of the Welsh Government COVID19 Technical Advisory Group. All other authors have declared no competing interests.

## Data Availability

The data used in this study are available in the SAIL Databank at Swansea University, Swansea, UK, but as restrictions apply they are not publicly available. All proposals to use SAIL data are subject to review by an independent Information Governance Review Panel (IGRP). Before any data can be accessed, approval must be given by the IGRP. The IGRP gives careful consideration to each project to ensure proper and appropriate use of SAIL data. When access has been granted, it is gained through a privacy protecting safe haven and remote access system referred to as the SAIL Gateway. SAIL has established an application process to be followed by anyone who would like to access data via SAIL at https://www.saildatabank.com/application-process.
