# Supplementary material for: COVID-19 vaccine uptake, effectiveness, and waning in 82,959 health care workers: A national prospective cohort study in Wales
Source: Vaccine. 2022 Feb 16;40(8):1180–9. doi: 10.1016/j.vaccine.2021.11.061 (PMC8760602; doi:10.1016/j.vaccine.2021.11.061)
Supplement: Supplementary data 1 [file mmc1.docx]

**COVID-19 Vaccine Uptake, Effectiveness, and Waning in 82,959 Health Care Workers: A national prospective cohort study in Wales**

# Supplementary Material

## Data sources

All people alive and resident in Wales were identified from the COVID-19 2020 (C20) cohort. The C20 cohort was derived from multiple data sources available within the SAIL Databank, and provided a population-scale, cleaned, and maintained e-cohort resource of demographic and associated details for the 3.2 million people alive and living in Wales from 1st January 2020. All health care workers were identified from all National Health Service (NHS) Wales organizations using national employment records managed by NHS Wales Shared Services Partnership (NWSSP), including details on specific roles and groups. Secondary care data covering 100%, and primary care data covering 80% of Wales (for those general practices that have opted in to share data with SAIL) were used to identify health service utilization. SARS-CoV-2 PCR tests covering all testing completed in Wales and on Welsh residents captured outside of Wales, including positive and negative results since the start of the pandemic. Pillar 1 and 2 data were used to identify any individual who had received testing, and if they had previously tested positive before the start of the vaccination programme as part of the exclusion criteria. Mortality data are available on all deaths occurring in Wales, and on Welsh residents captured outside of Wales from multiple sources including the Office for National Statistics (ONS) and Digital Health and Care Wales (DHCW). Vaccination data are collected as part of the Wales Immunisation System (WIS) and available on all vaccines occurring in Wales, and on Welsh residents captured outside of Wales.

**Table S1:** Data sources.

| SAIL acronym | Name | Description | Meta-data link |
| --- | --- | --- | --- |
| ADDD | Annual District Death Daily | Daily version of ADDE | <https://web.www.healthdatagateway.org/dataset/584bf8c8-d58f-44c6-9b65-e1611144fd54> |
| ADDE | Annual District Death Extract | Monthly register of all deaths relating to Welsh residents, including those that died out of Wales from the Office for National Statistics (ONS) | <https://web.www.healthdatagateway.org/dataset/15cf4241-abad-4dcc-95b0-8cd7c02be999> |
| CDDS | COVID-19 Consolidated Deaths | Daily update of all deaths recorded in any National Health Service (NHS) Wales linked system from Digital Health and Care Wales (DHCW) | <https://web.www.healthdatagateway.org/dataset/70e37f44-5c3e-4c83-a42a-89b3476d1d45> |
| CVVD | Covid Vaccination Dataset | Daily update of all appointments and vaccinations offered and or administered from the Welsh Immunization System (WIS) used to organize the delivery of the vaccination programme in Wales | <https://web.www.healthdatagateway.org/dataset/471f101c-a45e-4620-8710-be3036a46fba> |
| HWRA | Healthcare Workers Risk Assessment | Monthly record of all employed staff in secondary care organizations in Wales from NHS Wales Shared Services Partnership (NWSSP) | <https://web.www.healthdatagateway.org/dataset/ed131fee-0635-4613-a453-a383d5dd7cec> |
| PATD | COVID-19 Test results | Daily update of all pillar 1 and pillar 2 PCR tests administered and associated results for Welsh residents and those receiving a test in Wales | <https://web.www.healthdatagateway.org/dataset/f5f6d882-163d-4ef1-a53e-000fba409480> |
| PEDW | Patient Episode Dataset for Wales | Weekly update of all inpatient and day case activity undertaken in NHS Wales plus data on Welsh residents treated in English Trusts | <https://web.www.healthdatagateway.org/dataset/4c33a5d2-164c-41d7-9797-dc2b008cc852> |
| WDDS | Welsh Dispensing Dataset | Monthly updates of all prescriptions prescribed in Wales that are then dispensed in any pharmacy in the UK | <https://web.www.healthdatagateway.org/dataset/50ef6443-ed4b-40f9-97fb-1cfd53be6579> |
| WDSD | Welsh Demographic Service Dataset | Weekly update of all individuals registered with a Welsh general practice, including address and practice history | <https://web.www.healthdatagateway.org/dataset/8a8a5e90-b0c6-4839-bcd2-c69e6e8dca6d> |
| WLGP | Wales Longitudinal General Practice | Monthly update of all attendance and clinical information for SAIL submitting general practices (~80% national coverage) | <https://web.www.healthdatagateway.org/dataset/33fc3ffd-aa4c-4a16-a32f-0c900aaea3d2> |

## Measurement

**Table S2:** Definition of QCOVID items used for co-morbidity score.

| Item | Question/description | Values | Details |
| --- | --- | --- | --- |
| b2_82 | Have you been prescribed immunosuppressants prescribed by your GP | 0:false; 1:true | Prescribed four or more times in the previous 6 months |
| b2_leukolaba | Are you taking anti-leukotriene or long acting beta2-agonists (LABA)? | 0:false; 1:true | Prescribed four or more times in the previous 6 months |
| b2_prednisolone | Have you been prescribed oral steroids by your GP in the last 6 months? | 0:false; 1:true | oral predisolone containing preparations prescribed four or more times in the previous 6 months |
| b_AF | Do you have atrial fibrillation? | 0:false; 1:true |  |
| b_CCF | Do you have heart failure? | 0:false; 1:true |  |
| b_asthma | Do you have asthma? | 0:false; 1:true |  |
| b_bloodcancer | Have you a cancer of the blood or bone marrow such as leukaemia, myelodysplastic syndromes, lymphoma or myeloma and are at any stage of treatment? | 0:false; 1:true |  |
| b_cerebralpalsy | Do you have cerebral palsy? | 0:false; 1:true |  |
| b_chd | Do you have coronary heart disease? | 0:false; 1:true |  |
| b_cirrhosis | Do you have cirrhosis of the liver? | 0:false; 1:true |  |
| b_congenheart | Do you have congenital heart disease or have you had surgery for it in the past? | 0:false; 1:true | The categorization should be based either a Read code for congenital heart disease OR an HES OPCS code for surgery for congenital heart disease ever |
| b_copd | Do you have chronic obstructive pulmonary disease (COPD)? | 0:false; 1:true |  |
| b_dementia | Do you have dementia? | 0:false; 1:true |  |
| b_epilepsy | Do you have epilepsy? | 0:false; 1:true |  |
| b_fracture4 | Have you had a prior fracture of hip, wrist, spine or humerus? | 0:false; 1:true |  |
| b_neurorare | Do you have motor neurone disease, multiple sclerosis, myaesthenia, or Huntingtons's Chorea? | 0:false; 1:true |  |
| b_parkinsons | Do you have Parkinson’s disease? | 0:false; 1:true |  |
| b_pulmhyper | Do you have pulmonary hypertension or pulmonary fibrosis? | 0:false; 1:true |  |
| b_pulmrare | Do you have cystic fibrosis or bronchiectasis or alveolitis? | 0:false; 1:true |  |
| b_pvd | Do you have peripheral vascular disease? | 0:false; 1:true |  |
| b_ra_sle | Do you have rheumatoid arthritis or SLE? | 0:false; 1:true |  |
| b_respcancer | Do you have lung or oral cancer? | 0:false; 1:true |  |
| b_semi | Do you have severe mental illness? | 0:false; 1:true |  |
| b_sicklecelldisease | Do you have sickle cell disease or severe combined immune deficiency syndromes? | 0:false; 1:true |  |
| b_stroke | Have you had a stroke or TIA? | 0:false; 1:true |  |
| diabetes_cat | Do you have diabetes? | 0:none; 1:Type 1; 2:Type 2 |  |
| b_vte | Have you had a thrombosis or pulmonary embolus? | 0:false; 1:true |  |
| bmi | Body Mass Index | 15.0 to 47.0 | The most recently recorded patient BMI within the last 5 years. |
| chemocat | Have you had chemotherapy in the last 12 months? | 0:none; 1: Group A; 2: Group B; 3: Group C | Chemotherapy prescribed in preceding 12 months as recorded on the Systemic Anti Cancer Treatment (SACT) data. Chemotherapy classified into 3 cateogries (sheet 3) |
| homecat | what is your housing category - care home or homeless or neither? | 0: neither; 1: care home; 2: homeless | The most recently recorded accommodation status recorded on GP record |
| learncat | Do you have a learning disability or Down's Syndrome? | 0: neither; 1: learning disability; 2: Down's | The most recently recorded value. If some has a code both for learning disability and Downs, they should be coded as Downs |
| p_marrow6 | Have you had a bone marrow or stem cell transplant in the last 6 months? | 0:false; 1:true |  |
| p_radio6 | Have you had radiotherapy in the last 6 months? | 0:false; 1:true | coded as having radiotherapy in the preceding 6 months on either HES or RTDS |
| p_solidtransplant | Have you had a solid organ transplant (lung, liver, somach, pancreas, spleen, heart or thymus)? | 0:false; 1:true | coded as having solid organ transplant ever on HES |
| renalcat | Do you have kidney disease? | 1 to 6 | if ckd3==1 then code as 2; if ckd4==1 then replace as 3; if ckd5=1 then replace as 4; if ckd5=1 & dialysis in last 12 months replace as 5; if ckd5=1 & transplant ever code as 6 |

## Events

**Figure S1:** Weekly counts of all types of events. For disclosure purposes, counts between 1 and 9 replaced with a value of 5. Scales allowed to vary freely between panels.


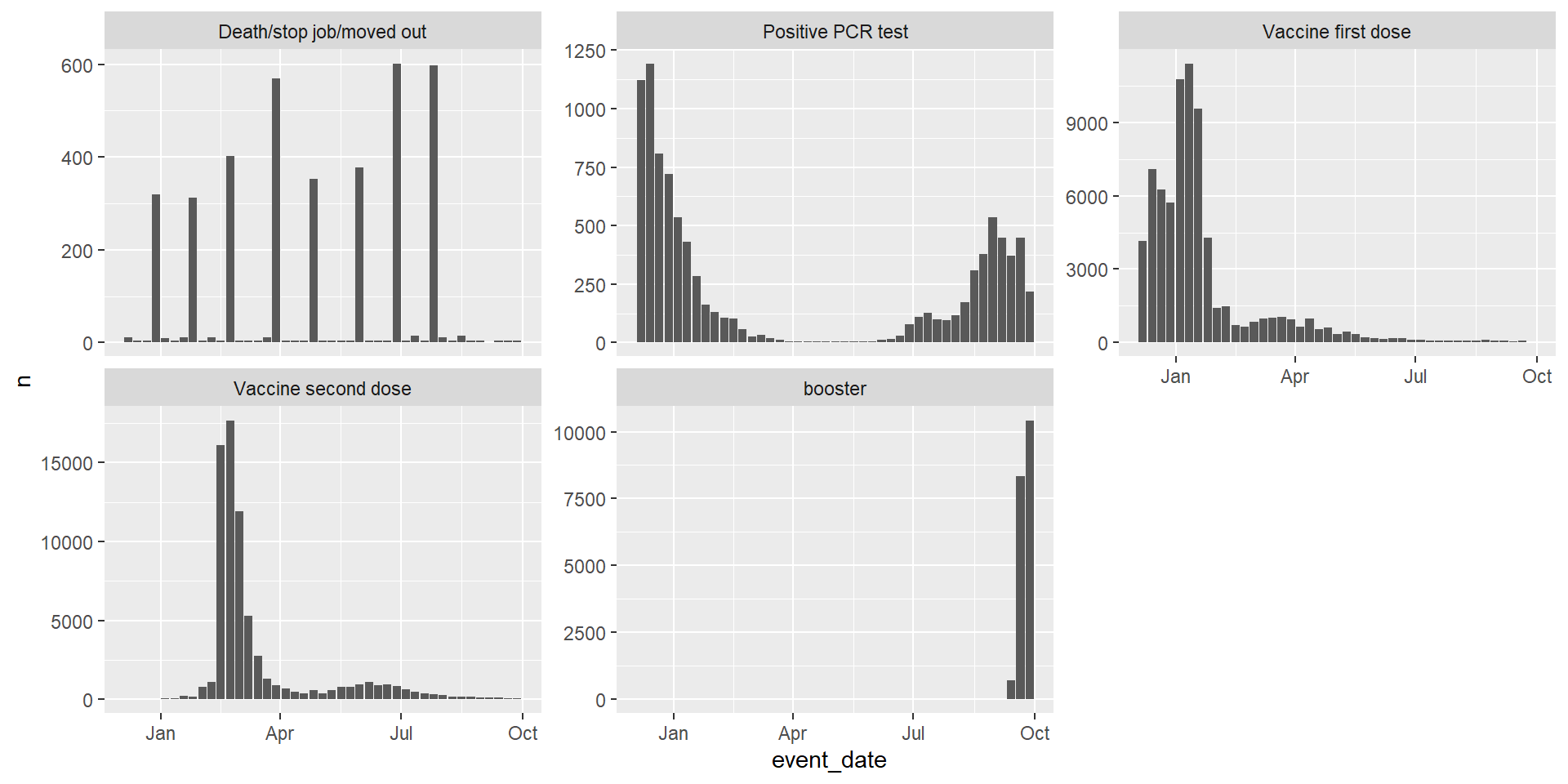


**Figure S2:** Timing of positive PCR test centered on date of first and second dose. For disclosure purposes, counts between 1 and 9 replaced with a value of 5.


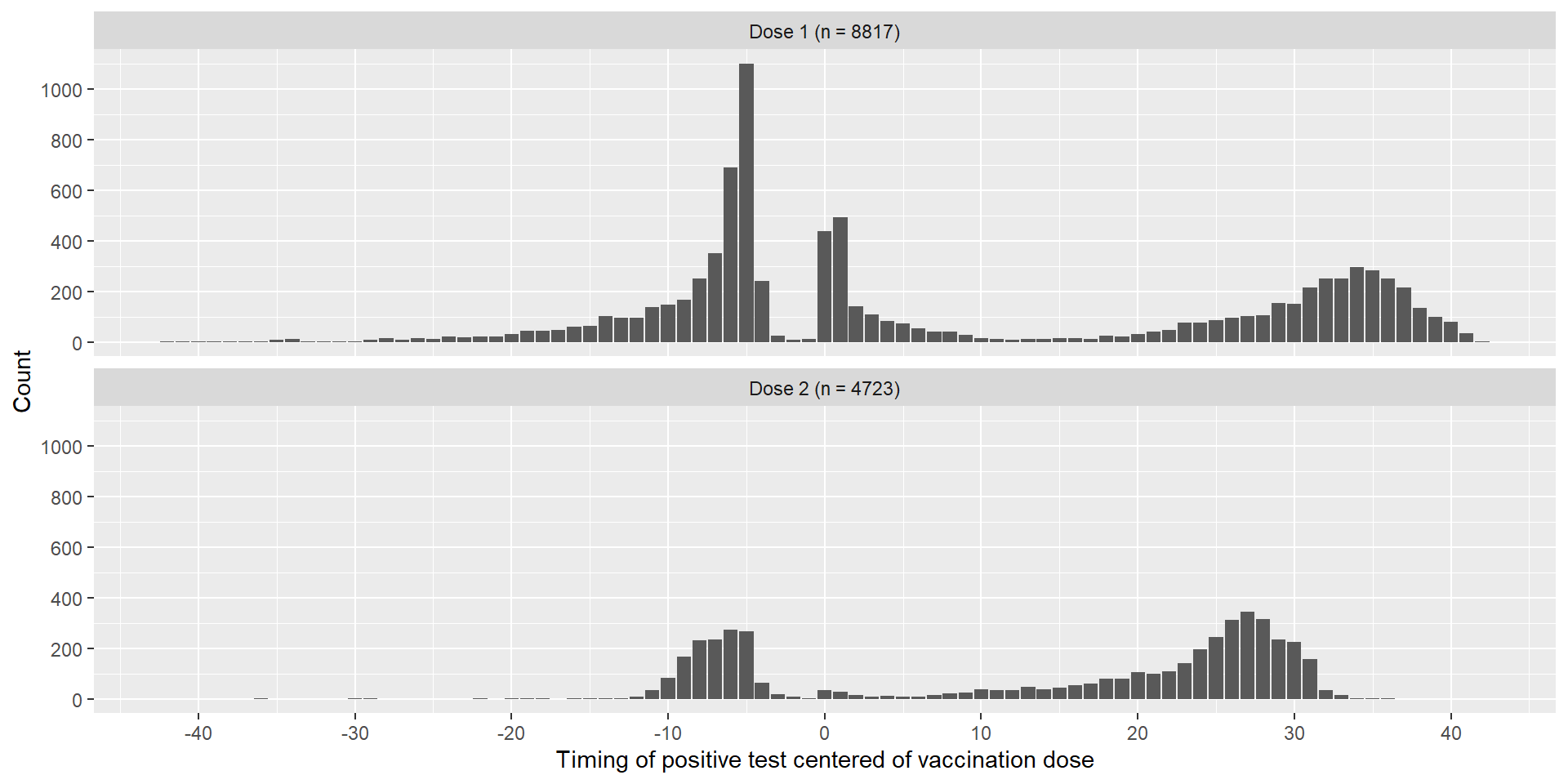


## Uptake

**Figure S3:** Empirical cumulative incidence curves for COVID-19 vaccine uptake by characteristics.


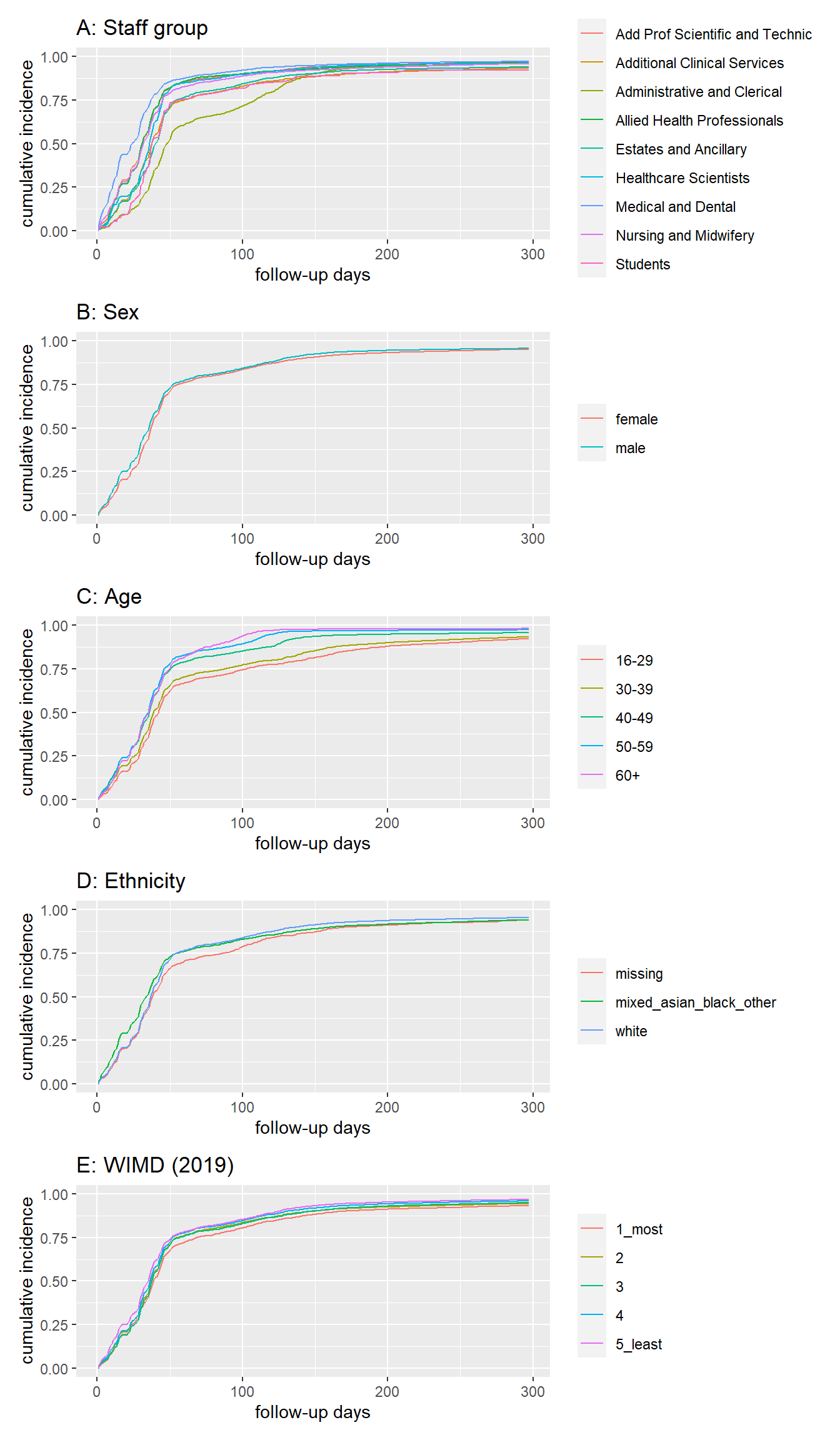


**
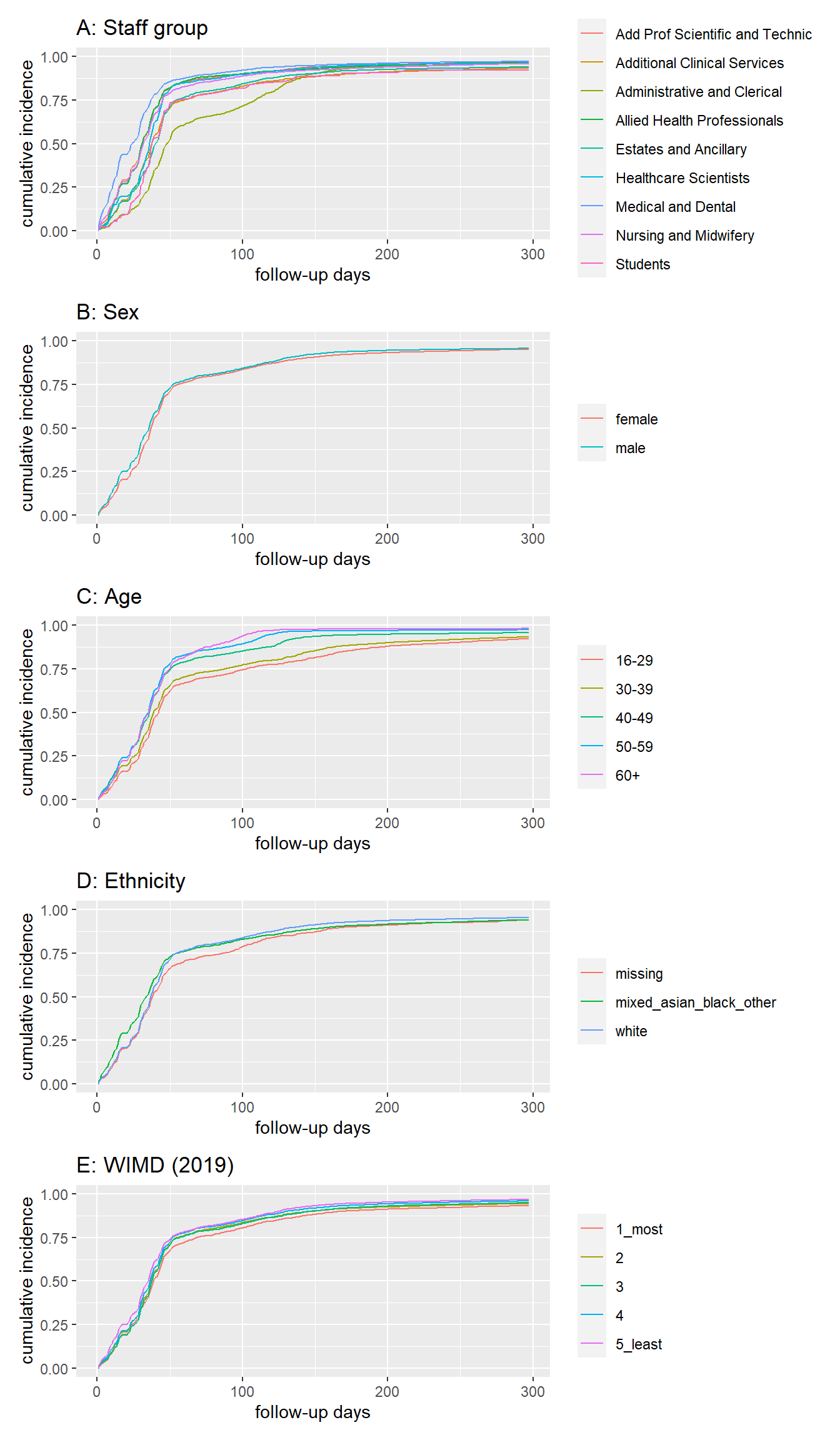
**

## Inverse propensity weights

**Figure S4:** Distribution of inverse propensity weights based on uptake of first dose by 25^th^ January 2021, by vaccination status at end of study.


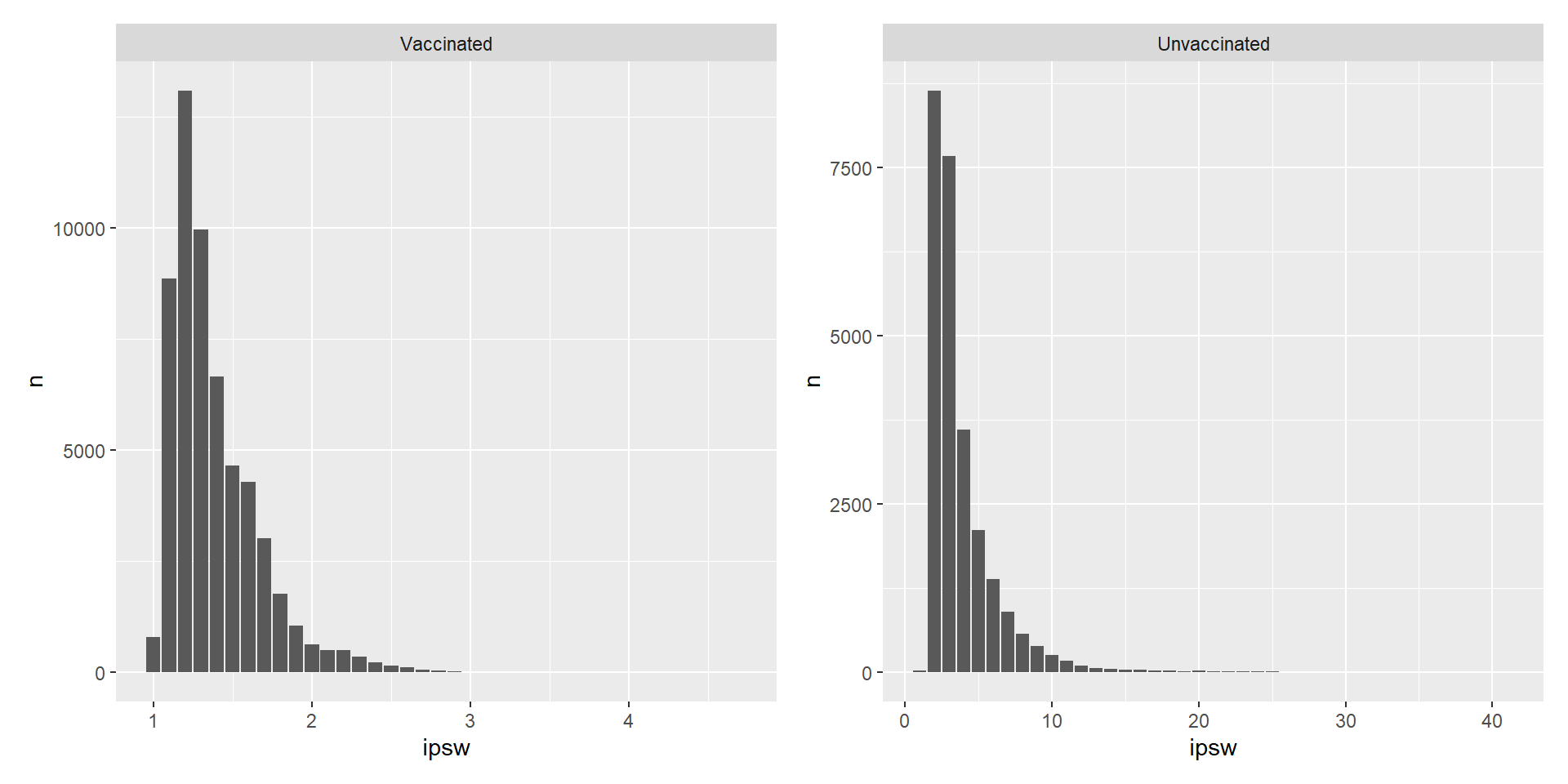


## Vaccine effectiveness

**Figure S5:** Comparison of odds ratios from the main analysis and the sensitivity analyses regarding discrete interval overall vaccine effectiveness.


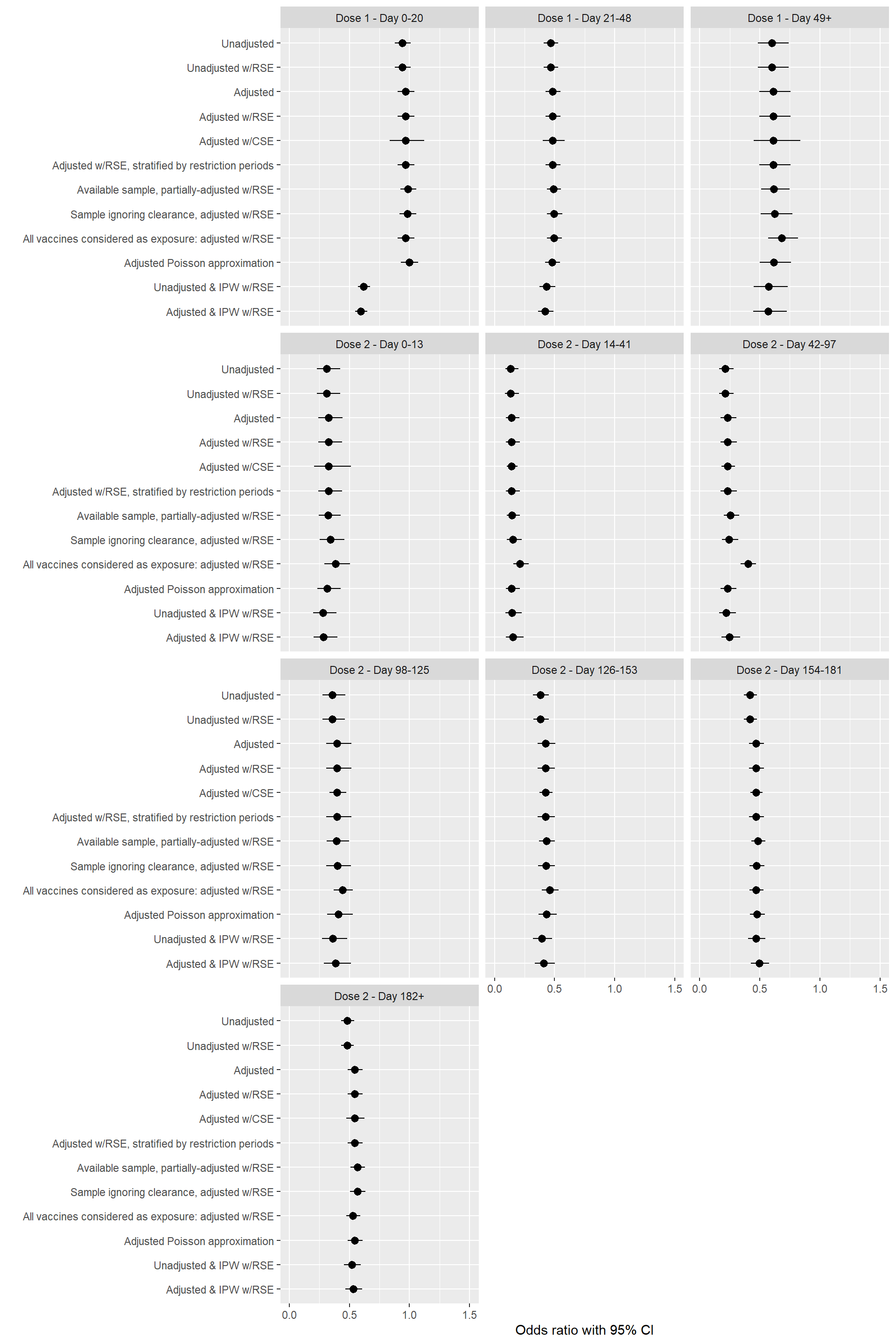


**Table S3:** Comparison of log hazard ratios and standard errors for overall vaccine effectiveness as shown in the main analysis in the manuscript and from sensitivity analyses A to D. (A) All health care workers with only partial adjustment. (B) Fully adjusted but ignore positive PCR tests prior to 7 December 2020. (C) Any COVID-19 vaccine is considered as the exposure (e.g. AZ, PF, MD). (D1) Inverse propensity weighted, adjusted estimates. (D2) Adjusted estimates with standard errors clustered by health board. (D3) Adjusted estimates with the baseline stratified by restriction periods in Wales. (D4) Poisson approximation of Cox proportional hazards model.

|  | Log HR |  |  |  |  |  |  |  |  |
| --- | --- | --- | --- | --- | --- | --- | --- | --- | --- |
| **Dose** | Main Unadjusted | Main Adjusted | A | B | C | D1 | D2 | D3 | D4 |
| **Unvaccinated** | 0.00 | 0.00 | 0.00 | 0.00 | 0.00 | 0.00 | 0.00 | 0.00 | 0.00 |
| **Dose 1** |  |  |  |  |  |  |  |  |  |
| Week 0-2 | -0.06 | -0.03 | -0.01 | -0.02 | -0.03 | -0.52 | -0.03 | -0.03 | 0.00 |
| Week 3-6 | -0.76 | -0.73 | -0.71 | -0.70 | -0.70 | -0.87 | -0.73 | -0.73 | -0.74 |
| Week 7+ | -0.51 | -0.49 | -0.48 | -0.47 | -0.38 | -0.57 | -0.49 | -0.49 | -0.49 |
| **Dose 2** |  |  |  |  |  |  |  |  |  |
| Week 0-1 | -1.17 | -1.12 | -1.13 | -1.08 | -0.96 | -1.26 | -1.12 | -1.12 | -1.16 |
| Week 2-5 | -2.01 | -1.96 | -1.92 | -1.88 | -1.55 | -1.88 | -1.96 | -1.96 | -1.95 |
| Week 6-13 | -1.54 | -1.46 | -1.36 | -1.41 | -0.91 | -1.40 | -1.46 | -1.46 | -1.47 |
| Week 14-17 | -1.02 | -0.92 | -0.94 | -0.92 | -0.82 | -0.96 | -0.92 | -0.92 | -0.90 |
| Week 18-21 | -0.96 | -0.85 | -0.84 | -0.85 | -0.78 | -0.89 | -0.85 | -0.85 | -0.83 |
| Week 22-25 | -0.87 | -0.76 | -0.72 | -0.75 | -0.75 | -0.70 | -0.76 | -0.76 | -0.74 |
| Week 26+ | -0.73 | -0.61 | -0.57 | -0.57 | -0.64 | -0.63 | -0.61 | -0.61 | -0.61 |
|  |  |  |  |  |  |  |  |  |  |
|  | Std. error |  |  |  |  |  |  |  |  |
| **Dose** | Main Unadjusted | Main Adjusted | A | B | C | D1 | D2 | D3 | D4 |
| **Unvaccinated** | 0.00 | 0.00 | 0.00 | 0.00 | 0.00 | 0.00 | 0.00 | 0.00 | 0.00 |
| **Dose 1** |  |  |  |  |  |  |  |  |  |
| Week 0-2 | 0.04 | 0.04 | 0.03 | 0.04 | 0.04 | 0.03 | 0.04 | 0.04 | 0.04 |
| Week 3-6 | 0.07 | 0.07 | 0.06 | 0.07 | 0.07 | 0.05 | 0.07 | 0.07 | 0.07 |
| Week 7+ | 0.11 | 0.11 | 0.10 | 0.11 | 0.09 | 0.08 | 0.11 | 0.11 | 0.11 |
| **Dose 2** |  |  |  |  |  |  |  |  |  |
| Week 0-1 | 0.16 | 0.16 | 0.14 | 0.15 | 0.14 | 0.12 | 0.16 | 0.16 | 0.16 |
| Week 2-5 | 0.20 | 0.20 | 0.18 | 0.20 | 0.15 | 0.13 | 0.20 | 0.20 | 0.20 |
| Week 6-13 | 0.14 | 0.14 | 0.12 | 0.14 | 0.08 | 0.09 | 0.14 | 0.14 | 0.14 |
| Week 14-17 | 0.13 | 0.13 | 0.12 | 0.13 | 0.09 | 0.08 | 0.13 | 0.13 | 0.13 |
| Week 18-21 | 0.09 | 0.09 | 0.08 | 0.09 | 0.08 | 0.06 | 0.09 | 0.09 | 0.09 |
| Week 22-25 | 0.07 | 0.07 | 0.06 | 0.07 | 0.06 | 0.04 | 0.07 | 0.07 | 0.07 |
| Week 26+ | 0.06 | 0.06 | 0.05 | 0.06 | 0.06 | 0.04 | 0.06 | 0.06 | 0.06 |

**Table S4:** Unadjusted and adjusted log hazard ratios for characteristic vaccine effectiveness against positive PCR test. Within each characteristic, hazard ratios are relative to those unvaccinated.

|  |  | **Unadjusted** |  |  |  |  | **Adjusted** |  |  |  |
| --- | --- | --- | --- | --- | --- | --- | --- | --- | --- | --- |
| **Characteristic / Dose** | | **Log HR** | **SE** | **Stat** | **p** |  | **Log HR** | **SE** | **Stat** | **p** |
| **Staff group** | |  |  |  |  |  |  |  |  |  |
| Additional Clinical Services | | | | | | | | | | |
|  | Dose 1 Wk 0-2 | 0.13 | 0.06 | 2.38 | 0.017 |  | 0.17 | 0.06 | 3.11 | 0.0018 |
|  | Dose 1 Wk 3-6 | -0.47 | 0.09 | -5.01 | 0.000 |  | -0.43 | 0.09 | -4.51 | 0.0000 |
|  | Dose 1 Wk 7+ | -0.50 | 0.17 | -3.00 | 0.003 |  | -0.47 | 0.17 | -2.85 | 0.0044 |
|  | Dose 2 Wk 0-1 | -0.84 | 0.22 | -3.90 | 0.000 |  | -0.80 | 0.22 | -3.69 | 0.0002 |
|  | Dose 2 Wk 2-5 | -1.89 | 0.31 | -6.11 | 0.000 |  | -1.85 | 0.31 | -6.03 | 0.0000 |
|  | Dose 2 Wk 6-13 | -1.44 | 0.23 | -6.23 | 0.000 |  | -1.42 | 0.23 | -6.15 | 0.0000 |
|  | Dose 2 Wk 14-17 | -0.76 | 0.17 | -4.45 | 0.000 |  | -0.69 | 0.17 | -4.05 | 0.0001 |
|  | Dose 2 Wk 18-21 | -1.06 | 0.12 | -8.82 | 0.000 |  | -0.97 | 0.12 | -8.09 | 0.0000 |
|  | Dose 2 Wk 22-25 | -0.80 | 0.08 | -9.66 | 0.000 |  | -0.71 | 0.08 | -8.49 | 0.0000 |
|  | Dose 2 Wk 26+ | -0.80 | 0.07 | -11.66 | 0.000 |  | -0.68 | 0.07 | -9.77 | 0.0000 |
| Nursing and Midwifery Registered | | | | | | | | | | |
|  | Dose 1 Wk 0-2 | -0.07 | 0.06 | -1.24 | 0.214 |  | -0.05 | 0.06 | -0.87 | 0.3840 |
|  | Dose 1 Wk 3-6 | -0.73 | 0.10 | -7.23 | 0.000 |  | -0.70 | 0.10 | -6.95 | 0.0000 |
|  | Dose 1 Wk 7+ | -0.61 | 0.18 | -3.35 | 0.001 |  | -0.58 | 0.18 | -3.19 | 0.0014 |
|  | Dose 2 Wk 0-1 | -1.30 | 0.27 | -4.82 | 0.000 |  | -1.26 | 0.27 | -4.68 | 0.0000 |
|  | Dose 2 Wk 2-5 | -1.98 | 0.34 | -5.67 | 0.000 |  | -1.95 | 0.34 | -5.60 | 0.0000 |
|  | Dose 2 Wk 6-13 | -1.41 | 0.27 | -5.14 | 0.000 |  | -1.39 | 0.27 | -5.11 | 0.0000 |
|  | Dose 2 Wk 14-17 | -1.25 | 0.23 | -5.53 | 0.000 |  | -1.19 | 0.23 | -5.24 | 0.0000 |
|  | Dose 2 Wk 18-21 | -0.89 | 0.12 | -7.53 | 0.000 |  | -0.81 | 0.12 | -6.85 | 0.0000 |
|  | Dose 2 Wk 22-25 | -0.88 | 0.09 | -9.84 | 0.000 |  | -0.79 | 0.09 | -8.83 | 0.0000 |
|  | Dose 2 Wk 26+ | -0.63 | 0.07 | -9.43 | 0.000 |  | -0.52 | 0.07 | -7.77 | 0.0000 |
| Admin | | | | | | | | | | |
|  | Dose 1 Wk 0-2 | -0.18 | 0.10 | -1.82 | 0.068 |  | -0.14 | 0.10 | -1.43 | 0.1520 |
|  | Dose 1 Wk 3-6 | -0.99 | 0.19 | -5.29 | 0.000 |  | -0.95 | 0.19 | -5.08 | 0.0000 |
|  | Dose 1 Wk 7+ | -0.01 | 0.22 | -0.06 | 0.948 |  | -0.02 | 0.22 | -0.09 | 0.9254 |
|  | Dose 2 Wk 0-1 | -1.95 | 0.50 | -3.84 | 0.000 |  | -1.92 | 0.50 | -3.80 | 0.0001 |
|  | Dose 2 Wk 2-5 | -1.45 | 0.31 | -4.74 | 0.000 |  | -1.46 | 0.31 | -4.76 | 0.0000 |
|  | Dose 2 Wk 6-13 | -1.15 | 0.18 | -6.31 | 0.000 |  | -1.15 | 0.18 | -6.29 | 0.0000 |
|  | Dose 2 Wk 14-17 | -0.74 | 0.20 | -3.80 | 0.000 |  | -0.66 | 0.20 | -3.37 | 0.0008 |
|  | Dose 2 Wk 18-21 | -0.74 | 0.14 | -5.27 | 0.000 |  | -0.64 | 0.14 | -4.59 | 0.0000 |
|  | Dose 2 Wk 22-25 | -0.68 | 0.10 | -6.67 | 0.000 |  | -0.58 | 0.10 | -5.63 | 0.0000 |
|  | Dose 2 Wk 26+ | -0.51 | 0.08 | -6.09 | 0.000 |  | -0.39 | 0.08 | -4.66 | 0.0000 |
| Estates and Ancillary | | | | | | | | | | |
|  | Dose 1 Wk 0-2 | -0.08 | 0.11 | -0.78 | 0.436 |  | -0.04 | 0.11 | -0.39 | 0.6998 |
|  | Dose 1 Wk 3-6 | -0.96 | 0.21 | -4.64 | 0.000 |  | -0.91 | 0.21 | -4.40 | 0.0000 |
|  | Dose 1 Wk 7+ | -1.04 | 0.41 | -2.52 | 0.012 |  | -1.03 | 0.41 | -2.50 | 0.0126 |
|  | Dose 2 Wk 0-1 | -0.99 | 0.42 | -2.39 | 0.017 |  | -0.95 | 0.42 | -2.29 | 0.0223 |
|  | Dose 2 Wk 2-5 | -3.03 | 1.00 | -3.00 | 0.003 |  | -3.00 | 1.00 | -2.98 | 0.0029 |
|  | Dose 2 Wk 6-13 | -1.84 | 0.51 | -3.63 | 0.000 |  | -1.82 | 0.51 | -3.62 | 0.0003 |
|  | Dose 2 Wk 14-17 | -1.68 | 0.42 | -3.98 | 0.000 |  | -1.60 | 0.42 | -3.79 | 0.0002 |
|  | Dose 2 Wk 18-21 | -1.28 | 0.22 | -5.92 | 0.000 |  | -1.18 | 0.22 | -5.49 | 0.0000 |
|  | Dose 2 Wk 22-25 | -0.75 | 0.13 | -5.95 | 0.000 |  | -0.64 | 0.13 | -5.11 | 0.0000 |
|  | Dose 2 Wk 26+ | -0.95 | 0.11 | -8.92 | 0.000 |  | -0.83 | 0.11 | -7.74 | 0.0000 |
| Medical and Dental | | | | | | | | | | |
|  | Dose 1 Wk 0-2 | -0.23 | 0.12 | -1.94 | 0.052 |  | -0.21 | 0.12 | -1.74 | 0.0821 |
|  | Dose 1 Wk 3-6 | -1.17 | 0.23 | -5.14 | 0.000 |  | -1.14 | 0.23 | -4.99 | 0.0000 |
|  | Dose 1 Wk 7+ | -0.45 | 0.31 | -1.45 | 0.147 |  | -0.41 | 0.31 | -1.31 | 0.1905 |
|  | Dose 2 Wk 0-1 | -1.02 | 0.46 | -2.22 | 0.027 |  | -0.97 | 0.46 | -2.11 | 0.0346 |
|  | Dose 2 Wk 2-5 | -2.06 | 0.71 | -2.88 | 0.004 |  | -2.02 | 0.71 | -2.83 | 0.0047 |
|  | Dose 2 Wk 6-13 | -2.63 | 1.00 | -2.64 | 0.008 |  | -2.61 | 1.00 | -2.62 | 0.0089 |
|  | Dose 2 Wk 14-17 | -2.15 | 0.71 | -2.99 | 0.003 |  | -2.09 | 0.71 | -2.92 | 0.0035 |
|  | Dose 2 Wk 18-21 | -1.02 | 0.24 | -4.36 | 0.000 |  | -0.94 | 0.24 | -4.02 | 0.0001 |
|  | Dose 2 Wk 22-25 | -0.97 | 0.18 | -5.52 | 0.000 |  | -0.88 | 0.18 | -5.03 | 0.0000 |
|  | Dose 2 Wk 26+ | -0.58 | 0.11 | -5.32 | 0.000 |  | -0.48 | 0.11 | -4.31 | 0.0000 |
| Allied Health Professionals | | | | | | | | | | |
|  | Dose 1 Wk 0-2 | -0.08 | 0.12 | -0.68 | 0.498 |  | -0.07 | 0.12 | -0.56 | 0.5756 |
|  | Dose 1 Wk 3-6 | -0.98 | 0.23 | -4.23 | 0.000 |  | -0.96 | 0.23 | -4.12 | 0.0000 |
|  | Dose 1 Wk 7+ | -1.41 | 0.58 | -2.42 | 0.015 |  | -1.39 | 0.58 | -2.39 | 0.0170 |
|  | Dose 2 Wk 0-1 | -1.15 | 0.51 | -2.25 | 0.024 |  | -1.12 | 0.51 | -2.19 | 0.0286 |
|  | Dose 2 Wk 2-5 | -13.60 | 226.38 | -131.47 | 0.000 |  | -13.58 | 227.47 | -133.69 | 0.0000 |
|  | Dose 2 Wk 6-13 | -1.59 | 0.58 | -2.68 | 0.007 |  | -1.57 | 0.58 | -2.65 | 0.0080 |
|  | Dose 2 Wk 14-17 | -1.58 | 0.51 | -3.10 | 0.002 |  | -1.51 | 0.51 | -2.97 | 0.0030 |
|  | Dose 2 Wk 18-21 | -0.67 | 0.20 | -3.36 | 0.001 |  | -0.59 | 0.20 | -2.97 | 0.0030 |
|  | Dose 2 Wk 22-25 | -0.85 | 0.16 | -5.30 | 0.000 |  | -0.76 | 0.16 | -4.77 | 0.0000 |
|  | Dose 2 Wk 26+ | -0.69 | 0.11 | -6.11 | 0.000 |  | -0.59 | 0.11 | -5.22 | 0.0000 |
| Technical Staff | |  |  |  |  |  |  |  |  |  |
|  | Dose 1 Wk 0-2 | -0.15 | 0.19 | -0.81 | 0.420 |  | -0.13 | 0.19 | -0.70 | 0.4813 |
|  | Dose 1 Wk 3-6 | -1.22 | 0.39 | -3.14 | 0.002 |  | -1.19 | 0.39 | -3.06 | 0.0022 |
|  | Dose 1 Wk 7+ | -0.05 | 0.46 | -0.10 | 0.917 |  | 0.00 | 0.46 | 0.00 | 0.9978 |
|  | Dose 2 Wk 0-1 | -1.73 | 1.01 | -1.72 | 0.086 |  | -1.69 | 1.01 | -1.68 | 0.0926 |
|  | Dose 2 Wk 2-5 | -13.37 | 310.39 | -106.39 | 0.000 |  | -13.35 | 311.79 | -107.18 | 0.0000 |
|  | Dose 2 Wk 6-13 | -1.16 | 0.72 | -1.60 | 0.109 |  | -1.13 | 0.72 | -1.57 | 0.1160 |
|  | Dose 2 Wk 14-17 | -0.95 | 0.59 | -1.60 | 0.109 |  | -0.88 | 0.59 | -1.50 | 0.1342 |
|  | Dose 2 Wk 18-21 | -0.57 | 0.29 | -1.95 | 0.051 |  | -0.49 | 0.29 | -1.68 | 0.0922 |
|  | Dose 2 Wk 22-25 | -0.86 | 0.25 | -3.51 | 0.000 |  | -0.78 | 0.25 | -3.16 | 0.0016 |
|  | Dose 2 Wk 26+ | -0.26 | 0.15 | -1.81 | 0.070 |  | -0.16 | 0.15 | -1.12 | 0.2612 |
| Healthcare Scientists | | | | | | | | | | |
|  | Dose 1 Wk 0-2 | -1.27 | 0.46 | -2.75 | 0.006 |  | -1.25 | 0.46 | -2.70 | 0.0070 |
|  | Dose 1 Wk 3-6 | -2.33 | 1.01 | -2.31 | 0.021 |  | -2.29 | 1.01 | -2.27 | 0.0232 |
|  | Dose 1 Wk 7+ | -0.72 | 1.01 | -0.71 | 0.477 |  | -0.70 | 1.01 | -0.69 | 0.4912 |
|  | Dose 2 Wk 0-1 | -13.08 | 443.22 | -93.25 | 0.000 |  | -13.04 | 444.12 | -93.49 | 0.0000 |
|  | Dose 2 Wk 2-5 | -13.26 | 435.42 | -85.86 | 0.000 |  | -13.24 | 437.84 | -86.47 | 0.0000 |
|  | Dose 2 Wk 6-13 | -0.62 | 0.72 | -0.86 | 0.389 |  | -0.59 | 0.72 | -0.83 | 0.4082 |
|  | Dose 2 Wk 14-17 | -0.26 | 0.52 | -0.50 | 0.615 |  | -0.19 | 0.52 | -0.37 | 0.7112 |
|  | Dose 2 Wk 18-21 | -0.84 | 0.43 | -1.95 | 0.052 |  | -0.76 | 0.43 | -1.76 | 0.0776 |
|  | Dose 2 Wk 22-25 | -0.68 | 0.30 | -2.30 | 0.022 |  | -0.59 | 0.30 | -2.00 | 0.0451 |
|  | Dose 2 Wk 26+ | -0.56 | 0.22 | -2.55 | 0.011 |  | -0.46 | 0.22 | -2.09 | 0.0365 |
| Students | | | | | | | | | | |
|  | Dose 1 Wk 0-2 | -12.97 | 440.16 | -44.93 | 0.000 |  | -12.97 | 441.33 | -44.92 | 0.0000 |
|  | Dose 1 Wk 3-6 | 0.89 | 0.64 | 1.38 | 0.168 |  | 0.90 | 0.64 | 1.39 | 0.1630 |
|  | Dose 1 Wk 7+ | 1.79 | 0.76 | 2.23 | 0.026 |  | 1.79 | 0.76 | 2.23 | 0.0259 |
|  | Dose 2 Wk 0-1 | -13.69 | 1531.88 | -44.06 | 0.000 |  | -13.66 | 1538.16 | -44.25 | 0.0000 |
|  | Dose 2 Wk 2-5 | 0.70 | 1.04 | 0.68 | 0.499 |  | 0.73 | 1.04 | 0.70 | 0.4857 |
|  | Dose 2 Wk 6-13 | -13.65 | 1128.13 | -35.13 | 0.000 |  | -13.64 | 1132.52 | -35.04 | 0.0000 |
|  | Dose 2 Wk 14-17 | -13.71 | 1093.84 | -44.13 | 0.000 |  | -13.66 | 1096.00 | -43.48 | 0.0000 |
|  | Dose 2 Wk 18-21 | -13.69 | 714.31 | -47.01 | 0.000 |  | -13.62 | 715.24 | -46.76 | 0.0000 |
|  | Dose 2 Wk 22-25 | -0.54 | 0.76 | -0.71 | 0.476 |  | -0.45 | 0.76 | -0.59 | 0.5543 |
|  | Dose 2 Wk 26+ | -1.90 | 1.04 | -1.82 | 0.068 |  | -1.81 | 1.04 | -1.73 | 0.0830 |
| **Sex** | | | | | | | | | | |
| Female | | | | | | | | | | |
|  | Dose 1 Wk 0-2 | -0.06 | 0.04 | -1.67 | 0.094 |  | -0.04 | 0.04 | -0.97 | 0.3328 |
|  | Dose 1 Wk 3-6 | -0.71 | 0.07 | -10.72 | 0.000 |  | -0.68 | 0.07 | -10.17 | 0.0000 |
|  | Dose 1 Wk 7+ | -0.45 | 0.11 | -4.26 | 0.000 |  | -0.43 | 0.11 | -4.08 | 0.0000 |
|  | Dose 2 Wk 0-1 | -1.16 | 0.16 | -7.30 | 0.000 |  | -1.11 | 0.16 | -7.05 | 0.0000 |
|  | Dose 2 Wk 2-5 | -1.80 | 0.19 | -9.46 | 0.000 |  | -1.76 | 0.19 | -9.28 | 0.0000 |
|  | Dose 2 Wk 6-13 | -1.43 | 0.14 | -10.39 | 0.000 |  | -1.36 | 0.14 | -9.89 | 0.0000 |
|  | Dose 2 Wk 14-17 | -0.90 | 0.12 | -7.32 | 0.000 |  | -0.79 | 0.12 | -6.47 | 0.0000 |
|  | Dose 2 Wk 18-21 | -0.95 | 0.09 | -11.39 | 0.000 |  | -0.84 | 0.09 | -10.06 | 0.0000 |
|  | Dose 2 Wk 22-25 | -0.82 | 0.06 | -13.15 | 0.000 |  | -0.71 | 0.06 | -11.16 | 0.0000 |
|  | Dose 2 Wk 26+ | -0.69 | 0.05 | -12.74 | 0.000 |  | -0.56 | 0.06 | -10.14 | 0.0000 |
| Male | | | | | | | | | | |
|  | Dose 1 Wk 0-2 | 0.04 | 0.07 | 0.55 | 0.581 |  | 0.07 | 0.07 | 1.06 | 0.2876 |
|  | Dose 1 Wk 3-6 | -0.91 | 0.13 | -6.99 | 0.000 |  | -0.86 | 0.13 | -6.63 | 0.0000 |
|  | Dose 1 Wk 7+ | -0.77 | 0.24 | -3.21 | 0.001 |  | -0.73 | 0.24 | -3.01 | 0.0026 |
|  | Dose 2 Wk 0-1 | -1.25 | 0.31 | -4.05 | 0.000 |  | -1.20 | 0.31 | -3.89 | 0.0001 |
|  | Dose 2 Wk 2-5 | -3.21 | 0.71 | -4.53 | 0.000 |  | -3.16 | 0.71 | -4.45 | 0.0000 |
|  | Dose 2 Wk 6-13 | -1.43 | 0.25 | -5.67 | 0.000 |  | -1.34 | 0.25 | -5.34 | 0.0000 |
|  | Dose 2 Wk 14-17 | -1.91 | 0.33 | -5.81 | 0.000 |  | -1.81 | 0.33 | -5.50 | 0.0000 |
|  | Dose 2 Wk 18-21 | -0.96 | 0.14 | -7.16 | 0.000 |  | -0.85 | 0.14 | -6.33 | 0.0000 |
|  | Dose 2 Wk 22-25 | -0.90 | 0.10 | -9.21 | 0.000 |  | -0.79 | 0.10 | -8.02 | 0.0000 |
|  | Dose 2 Wk 26+ | -0.71 | 0.07 | -9.68 | 0.000 |  | -0.58 | 0.08 | -7.79 | 0.0000 |
| **Age** | | | | | | | | | | |
| 16-29 | | | | | | | | | | |
|  | Dose 1 Wk 0-2 | 0.02 | 0.07 | 0.33 | 0.744 |  | 0.05 | 0.07 | 0.72 | 0.4704 |
|  | Dose 1 Wk 3-6 | -0.80 | 0.13 | -6.14 | 0.000 |  | -0.77 | 0.13 | -5.88 | 0.0000 |
|  | Dose 1 Wk 7+ | -0.62 | 0.19 | -3.23 | 0.001 |  | -0.60 | 0.19 | -3.13 | 0.0017 |
|  | Dose 2 Wk 0-1 | -0.90 | 0.25 | -3.62 | 0.000 |  | -0.87 | 0.25 | -3.48 | 0.0005 |
|  | Dose 2 Wk 2-5 | -3.19 | 0.58 | -5.47 | 0.000 |  | -3.14 | 0.58 | -5.39 | 0.0000 |
|  | Dose 2 Wk 6-13 | -1.58 | 0.22 | -7.07 | 0.000 |  | -1.52 | 0.22 | -6.77 | 0.0000 |
|  | Dose 2 Wk 14-17 | -0.89 | 0.21 | -4.19 | 0.000 |  | -0.85 | 0.21 | -4.03 | 0.0001 |
|  | Dose 2 Wk 18-21 | -0.75 | 0.13 | -5.79 | 0.000 |  | -0.71 | 0.13 | -5.49 | 0.0000 |
|  | Dose 2 Wk 22-25 | -0.66 | 0.09 | -6.95 | 0.000 |  | -0.62 | 0.10 | -6.55 | 0.0000 |
|  | Dose 2 Wk 26+ | -0.94 | 0.08 | -10.93 | 0.000 |  | -0.89 | 0.09 | -10.31 | 0.0000 |
| 30-39 | | | | | | | | | | |
|  | Dose 1 Wk 0-2 | -0.14 | 0.07 | -2.01 | 0.045 |  | -0.13 | 0.07 | -1.81 | 0.0695 |
|  | Dose 1 Wk 3-6 | -0.74 | 0.11 | -6.44 | 0.000 |  | -0.72 | 0.12 | -6.27 | 0.0000 |
|  | Dose 1 Wk 7+ | -0.53 | 0.18 | -3.01 | 0.003 |  | -0.52 | 0.18 | -2.93 | 0.0034 |
|  | Dose 2 Wk 0-1 | -1.25 | 0.27 | -4.60 | 0.000 |  | -1.22 | 0.27 | -4.50 | 0.0000 |
|  | Dose 2 Wk 2-5 | -1.41 | 0.24 | -5.76 | 0.000 |  | -1.37 | 0.24 | -5.58 | 0.0000 |
|  | Dose 2 Wk 6-13 | -1.42 | 0.20 | -6.96 | 0.000 |  | -1.35 | 0.20 | -6.63 | 0.0000 |
|  | Dose 2 Wk 14-17 | -1.08 | 0.21 | -5.07 | 0.000 |  | -1.04 | 0.21 | -4.87 | 0.0000 |
|  | Dose 2 Wk 18-21 | -0.76 | 0.12 | -6.32 | 0.000 |  | -0.72 | 0.12 | -6.01 | 0.0000 |
|  | Dose 2 Wk 22-25 | -0.53 | 0.08 | -6.28 | 0.000 |  | -0.50 | 0.08 | -5.83 | 0.0000 |
|  | Dose 2 Wk 26+ | -0.47 | 0.07 | -6.75 | 0.000 |  | -0.43 | 0.07 | -6.18 | 0.0000 |
| 40-49 | | | | | | | | | | |
|  | Dose 1 Wk 0-2 | -0.06 | 0.07 | -0.88 | 0.380 |  | -0.06 | 0.07 | -0.86 | 0.3872 |
|  | Dose 1 Wk 3-6 | -0.69 | 0.11 | -6.10 | 0.000 |  | -0.69 | 0.11 | -6.04 | 0.0000 |
|  | Dose 1 Wk 7+ | -0.52 | 0.21 | -2.51 | 0.012 |  | -0.51 | 0.21 | -2.43 | 0.0151 |
|  | Dose 2 Wk 0-1 | -1.20 | 0.30 | -4.03 | 0.000 |  | -1.20 | 0.30 | -4.00 | 0.0001 |
|  | Dose 2 Wk 2-5 | -1.90 | 0.39 | -4.89 | 0.000 |  | -1.88 | 0.39 | -4.82 | 0.0000 |
|  | Dose 2 Wk 6-13 | -1.09 | 0.21 | -5.02 | 0.000 |  | -1.01 | 0.21 | -4.64 | 0.0000 |
|  | Dose 2 Wk 14-17 | -0.73 | 0.18 | -3.96 | 0.000 |  | -0.69 | 0.18 | -3.72 | 0.0002 |
|  | Dose 2 Wk 18-21 | -0.96 | 0.13 | -7.58 | 0.000 |  | -0.93 | 0.13 | -7.32 | 0.0000 |
|  | Dose 2 Wk 22-25 | -0.83 | 0.09 | -9.00 | 0.000 |  | -0.80 | 0.09 | -8.66 | 0.0000 |
|  | Dose 2 Wk 26+ | -0.36 | 0.07 | -5.34 | 0.000 |  | -0.33 | 0.07 | -4.92 | 0.0000 |
| 50-59 | | | | | | | | | | |
|  | Dose 1 Wk 0-2 | 0.07 | 0.06 | 1.11 | 0.265 |  | 0.07 | 0.06 | 1.05 | 0.2922 |
|  | Dose 1 Wk 3-6 | -0.73 | 0.12 | -6.33 | 0.000 |  | -0.73 | 0.12 | -6.31 | 0.0000 |
|  | Dose 1 Wk 7+ | -0.24 | 0.20 | -1.17 | 0.242 |  | -0.24 | 0.20 | -1.16 | 0.2474 |
|  | Dose 2 Wk 0-1 | -1.38 | 0.33 | -4.21 | 0.000 |  | -1.38 | 0.33 | -4.20 | 0.0000 |
|  | Dose 2 Wk 2-5 | -2.12 | 0.46 | -4.61 | 0.000 |  | -2.11 | 0.46 | -4.58 | 0.0000 |
|  | Dose 2 Wk 6-13 | -1.71 | 0.36 | -4.73 | 0.000 |  | -1.65 | 0.37 | -4.56 | 0.0000 |
|  | Dose 2 Wk 14-17 | -1.23 | 0.23 | -5.48 | 0.000 |  | -1.19 | 0.23 | -5.30 | 0.0000 |
|  | Dose 2 Wk 18-21 | -1.01 | 0.13 | -7.68 | 0.000 |  | -0.98 | 0.13 | -7.46 | 0.0000 |
|  | Dose 2 Wk 22-25 | -0.96 | 0.10 | -9.98 | 0.000 |  | -0.93 | 0.10 | -9.66 | 0.0000 |
|  | Dose 2 Wk 26+ | -0.78 | 0.07 | -10.59 | 0.000 |  | -0.76 | 0.07 | -10.25 | 0.0000 |
| 60+ | | | | | | | | | | |
|  | Dose 1 Wk 0-2 | 0.03 | 0.11 | 0.31 | 0.759 |  | 0.03 | 0.11 | 0.25 | 0.7988 |
|  | Dose 1 Wk 3-6 | -0.59 | 0.19 | -3.14 | 0.002 |  | -0.59 | 0.19 | -3.15 | 0.0016 |
|  | Dose 1 Wk 7+ | -0.54 | 0.41 | -1.30 | 0.195 |  | -0.53 | 0.41 | -1.28 | 0.2007 |
|  | Dose 2 Wk 0-1 | -0.94 | 0.46 | -2.09 | 0.036 |  | -0.95 | 0.46 | -2.10 | 0.0357 |
|  | Dose 2 Wk 2-5 | -1.84 | 0.72 | -2.54 | 0.011 |  | -1.84 | 0.72 | -2.54 | 0.0111 |
|  | Dose 2 Wk 6-13 | -2.34 | 1.01 | -2.35 | 0.019 |  | -2.29 | 1.01 | -2.31 | 0.0211 |
|  | Dose 2 Wk 14-17 | -1.56 | 0.46 | -3.44 | 0.001 |  | -1.53 | 0.46 | -3.36 | 0.0008 |
|  | Dose 2 Wk 18-21 | -1.16 | 0.23 | -5.14 | 0.000 |  | -1.14 | 0.23 | -5.03 | 0.0000 |
|  | Dose 2 Wk 22-25 | -1.04 | 0.16 | -6.51 | 0.000 |  | -1.01 | 0.16 | -6.33 | 0.0000 |
|  | Dose 2 Wk 26+ | -0.78 | 0.11 | -7.03 | 0.000 |  | -0.76 | 0.11 | -6.82 | 0.0000 |
| **Ethnicity** | | | | | | | | | | |
| White | | | | | | | | | | |
|  | Dose 1 Wk 0-2 | -0.04 | 0.04 | -1.03 | 0.302 |  | -0.01 | 0.04 | -0.26 | 0.7915 |
|  | Dose 1 Wk 3-6 | -0.73 | 0.06 | -11.52 | 0.000 |  | -0.69 | 0.06 | -10.90 | 0.0000 |
|  | Dose 1 Wk 7+ | -0.50 | 0.10 | -4.82 | 0.000 |  | -0.48 | 0.10 | -4.63 | 0.0000 |
|  | Dose 2 Wk 0-1 | -1.20 | 0.15 | -7.81 | 0.000 |  | -1.15 | 0.15 | -7.55 | 0.0000 |
|  | Dose 2 Wk 2-5 | -1.98 | 0.19 | -10.14 | 0.000 |  | -1.94 | 0.19 | -9.97 | 0.0000 |
|  | Dose 2 Wk 6-13 | -1.48 | 0.13 | -10.93 | 0.000 |  | -1.40 | 0.13 | -10.38 | 0.0000 |
|  | Dose 2 Wk 14-17 | -1.02 | 0.12 | -8.37 | 0.000 |  | -0.91 | 0.12 | -7.47 | 0.0000 |
|  | Dose 2 Wk 18-21 | -0.92 | 0.08 | -11.49 | 0.000 |  | -0.81 | 0.08 | -10.06 | 0.0000 |
|  | Dose 2 Wk 22-25 | -0.82 | 0.06 | -13.53 | 0.000 |  | -0.70 | 0.06 | -11.44 | 0.0000 |
|  | Dose 2 Wk 26+ | -0.67 | 0.05 | -12.70 | 0.000 |  | -0.54 | 0.05 | -10.00 | 0.0000 |
| Minority ethic | | | | | | | | | | |
|  | Dose 1 Wk 0-2 | -0.11 | 0.10 | -1.05 | 0.294 |  | -0.04 | 0.10 | -0.37 | 0.7113 |
|  | Dose 1 Wk 3-6 | -1.00 | 0.20 | -5.13 | 0.000 |  | -0.93 | 0.20 | -4.72 | 0.0000 |
|  | Dose 1 Wk 7+ | -0.60 | 0.31 | -1.95 | 0.051 |  | -0.52 | 0.31 | -1.69 | 0.0919 |
|  | Dose 2 Wk 0-1 | -1.78 | 0.58 | -3.07 | 0.002 |  | -1.70 | 0.58 | -2.92 | 0.0034 |
|  | Dose 2 Wk 2-5 | -2.06 | 0.58 | -3.52 | 0.000 |  | -1.98 | 0.58 | -3.38 | 0.0007 |
|  | Dose 2 Wk 6-13 | -1.11 | 0.32 | -3.42 | 0.001 |  | -1.02 | 0.32 | -3.16 | 0.0016 |
|  | Dose 2 Wk 14-17 | -1.33 | 0.39 | -3.41 | 0.001 |  | -1.21 | 0.39 | -3.12 | 0.0018 |
|  | Dose 2 Wk 18-21 | -1.52 | 0.25 | -6.03 | 0.000 |  | -1.39 | 0.25 | -5.49 | 0.0000 |
|  | Dose 2 Wk 22-25 | -1.27 | 0.17 | -7.59 | 0.000 |  | -1.13 | 0.17 | -6.75 | 0.0000 |
|  | Dose 2 Wk 26+ | -1.00 | 0.11 | -9.21 | 0.000 |  | -0.83 | 0.11 | -7.63 | 0.0000 |
| (missing) | | | | | | | | | | |
|  | Dose 1 Wk 0-2 | -0.23 | 0.36 | -0.63 | 0.529 |  | -0.17 | 0.36 | -0.48 | 0.6342 |
|  | Dose 1 Wk 3-6 | -0.81 | 0.59 | -1.35 | 0.176 |  | -0.75 | 0.59 | -1.25 | 0.2104 |
|  | Dose 1 Wk 7+ | -0.67 | 1.01 | -0.67 | 0.503 |  | -0.60 | 1.01 | -0.60 | 0.5517 |
|  | Dose 2 Wk 0-1 | 0.78 | 0.52 | 1.50 | 0.134 |  | 0.83 | 0.52 | 1.59 | 0.1120 |
|  | Dose 2 Wk 2-5 | -0.95 | 1.01 | -0.93 | 0.351 |  | -0.89 | 1.01 | -0.88 | 0.3791 |
|  | Dose 2 Wk 6-13 | -1.25 | 1.01 | -1.24 | 0.215 |  | -1.18 | 1.01 | -1.17 | 0.2425 |
|  | Dose 2 Wk 14-17 | -1.04 | 1.01 | -1.03 | 0.303 |  | -0.97 | 1.01 | -0.96 | 0.3376 |
|  | Dose 2 Wk 18-21 | -0.62 | 0.52 | -1.18 | 0.237 |  | -0.51 | 0.52 | -0.98 | 0.3282 |
|  | Dose 2 Wk 22-25 | -0.10 | 0.31 | -0.31 | 0.753 |  | 0.01 | 0.31 | 0.04 | 0.9657 |
|  | Dose 2 Wk 26+ | -0.58 | 0.29 | -2.01 | 0.045 |  | -0.42 | 0.29 | -1.45 | 0.1472 |
| **SES quintile** | | | | | | | | | | |
| 1st (Most deprived) | | | | | | | | | | |
|  | Dose 1 Wk 0-2 | -0.23 | 0.09 | -2.69 | 0.007 |  | -0.20 | 0.09 | -2.36 | 0.0182 |
|  | Dose 1 Wk 3-6 | -0.67 | 0.14 | -4.98 | 0.000 |  | -0.64 | 0.14 | -4.73 | 0.0000 |
|  | Dose 1 Wk 7+ | -0.75 | 0.25 | -2.94 | 0.003 |  | -0.73 | 0.25 | -2.87 | 0.0041 |
|  | Dose 2 Wk 0-1 | -1.01 | 0.31 | -3.23 | 0.001 |  | -0.97 | 0.31 | -3.10 | 0.0019 |
|  | Dose 2 Wk 2-5 | -1.96 | 0.42 | -4.63 | 0.000 |  | -1.92 | 0.42 | -4.53 | 0.0000 |
|  | Dose 2 Wk 6-13 | -1.43 | 0.27 | -5.23 | 0.000 |  | -1.34 | 0.27 | -4.92 | 0.0000 |
|  | Dose 2 Wk 14-17 | -1.02 | 0.25 | -4.13 | 0.000 |  | -0.93 | 0.25 | -3.76 | 0.0002 |
|  | Dose 2 Wk 18-21 | -1.08 | 0.16 | -6.94 | 0.000 |  | -0.98 | 0.16 | -6.30 | 0.0000 |
|  | Dose 2 Wk 22-25 | -0.90 | 0.11 | -8.24 | 0.000 |  | -0.79 | 0.11 | -7.26 | 0.0000 |
|  | Dose 2 Wk 26+ | -0.69 | 0.08 | -8.61 | 0.000 |  | -0.57 | 0.08 | -7.04 | 0.0000 |
| 2nd | | | | | | | | | | |
|  | Dose 1 Wk 0-2 | -0.05 | 0.07 | -0.73 | 0.468 |  | -0.03 | 0.07 | -0.42 | 0.6763 |
|  | Dose 1 Wk 3-6 | -0.75 | 0.13 | -5.94 | 0.000 |  | -0.72 | 0.13 | -5.71 | 0.0000 |
|  | Dose 1 Wk 7+ | -0.81 | 0.24 | -3.39 | 0.001 |  | -0.80 | 0.24 | -3.34 | 0.0008 |
|  | Dose 2 Wk 0-1 | -1.52 | 0.36 | -4.25 | 0.000 |  | -1.49 | 0.36 | -4.15 | 0.0000 |
|  | Dose 2 Wk 2-5 | -2.28 | 0.45 | -5.07 | 0.000 |  | -2.24 | 0.45 | -4.99 | 0.0000 |
|  | Dose 2 Wk 6-13 | -1.48 | 0.27 | -5.56 | 0.000 |  | -1.41 | 0.27 | -5.30 | 0.0000 |
|  | Dose 2 Wk 14-17 | -1.25 | 0.24 | -5.01 | 0.000 |  | -1.15 | 0.24 | -4.63 | 0.0000 |
|  | Dose 2 Wk 18-21 | -1.10 | 0.14 | -7.69 | 0.000 |  | -1.00 | 0.14 | -7.01 | 0.0000 |
|  | Dose 2 Wk 22-25 | -0.80 | 0.10 | -8.34 | 0.000 |  | -0.69 | 0.10 | -7.23 | 0.0000 |
|  | Dose 2 Wk 26+ | -0.62 | 0.07 | -8.54 | 0.000 |  | -0.51 | 0.07 | -6.88 | 0.0000 |
| 3rd | | | | | | | | | | |
|  | Dose 1 Wk 0-2 | 0.13 | 0.07 | 1.87 | 0.061 |  | 0.15 | 0.07 | 2.16 | 0.0310 |
|  | Dose 1 Wk 3-6 | -0.69 | 0.13 | -5.47 | 0.000 |  | -0.66 | 0.13 | -5.24 | 0.0000 |
|  | Dose 1 Wk 7+ | -0.59 | 0.22 | -2.71 | 0.007 |  | -0.59 | 0.22 | -2.71 | 0.0068 |
|  | Dose 2 Wk 0-1 | -1.03 | 0.29 | -3.64 | 0.000 |  | -1.00 | 0.28 | -3.54 | 0.0004 |
|  | Dose 2 Wk 2-5 | -1.70 | 0.34 | -5.03 | 0.000 |  | -1.67 | 0.34 | -4.96 | 0.0000 |
|  | Dose 2 Wk 6-13 | -1.41 | 0.26 | -5.55 | 0.000 |  | -1.35 | 0.26 | -5.33 | 0.0000 |
|  | Dose 2 Wk 14-17 | -0.98 | 0.22 | -4.53 | 0.000 |  | -0.88 | 0.22 | -4.07 | 0.0000 |
|  | Dose 2 Wk 18-21 | -0.87 | 0.13 | -6.54 | 0.000 |  | -0.77 | 0.13 | -5.78 | 0.0000 |
|  | Dose 2 Wk 22-25 | -0.81 | 0.10 | -8.43 | 0.000 |  | -0.71 | 0.10 | -7.30 | 0.0000 |
|  | Dose 2 Wk 26+ | -0.73 | 0.08 | -9.62 | 0.000 |  | -0.62 | 0.08 | -8.05 | 0.0000 |
| 4th | | | | | | | | | | |
|  | Dose 1 Wk 0-2 | -0.01 | 0.07 | -0.10 | 0.922 |  | 0.01 | 0.07 | 0.20 | 0.8441 |
|  | Dose 1 Wk 3-6 | -0.61 | 0.12 | -5.30 | 0.000 |  | -0.59 | 0.12 | -5.06 | 0.0000 |
|  | Dose 1 Wk 7+ | -0.20 | 0.18 | -1.13 | 0.258 |  | -0.18 | 0.18 | -0.99 | 0.3203 |
|  | Dose 2 Wk 0-1 | -1.09 | 0.29 | -3.83 | 0.000 |  | -1.06 | 0.29 | -3.72 | 0.0002 |
|  | Dose 2 Wk 2-5 | -1.73 | 0.34 | -4.96 | 0.000 |  | -1.70 | 0.34 | -4.90 | 0.0000 |
|  | Dose 2 Wk 6-13 | -1.17 | 0.23 | -4.96 | 0.000 |  | -1.11 | 0.23 | -4.74 | 0.0000 |
|  | Dose 2 Wk 14-17 | -1.01 | 0.22 | -4.77 | 0.000 |  | -0.92 | 0.22 | -4.33 | 0.0000 |
|  | Dose 2 Wk 18-21 | -0.84 | 0.13 | -6.58 | 0.000 |  | -0.74 | 0.13 | -5.79 | 0.0000 |
|  | Dose 2 Wk 22-25 | -0.83 | 0.10 | -8.72 | 0.000 |  | -0.72 | 0.10 | -7.57 | 0.0000 |
|  | Dose 2 Wk 26+ | -0.78 | 0.08 | -10.42 | 0.000 |  | -0.67 | 0.08 | -8.81 | 0.0000 |
| 5th (Least) | | | | | | | | | | |
|  | Dose 1 Wk 0-2 | -0.06 | 0.07 | -0.98 | 0.329 |  | -0.04 | 0.07 | -0.53 | 0.5949 |
|  | Dose 1 Wk 3-6 | -0.96 | 0.13 | -7.54 | 0.000 |  | -0.92 | 0.13 | -7.25 | 0.0000 |
|  | Dose 1 Wk 7+ | -0.31 | 0.19 | -1.63 | 0.103 |  | -0.28 | 0.19 | -1.47 | 0.1419 |
|  | Dose 2 Wk 0-1 | -1.21 | 0.29 | -4.19 | 0.000 |  | -1.17 | 0.29 | -4.05 | 0.0001 |
|  | Dose 2 Wk 2-5 | -2.23 | 0.42 | -5.40 | 0.000 |  | -2.19 | 0.42 | -5.31 | 0.0000 |
|  | Dose 2 Wk 6-13 | -1.64 | 0.27 | -6.14 | 0.000 |  | -1.59 | 0.27 | -5.93 | 0.0000 |
|  | Dose 2 Wk 14-17 | -0.91 | 0.20 | -4.49 | 0.000 |  | -0.82 | 0.20 | -4.03 | 0.0001 |
|  | Dose 2 Wk 18-21 | -0.88 | 0.13 | -6.98 | 0.000 |  | -0.78 | 0.13 | -6.16 | 0.0000 |
|  | Dose 2 Wk 22-25 | -0.82 | 0.09 | -8.80 | 0.000 |  | -0.72 | 0.09 | -7.66 | 0.0000 |
|  | Dose 2 Wk 26+ | -0.60 | 0.07 | -8.54 | 0.000 |  | -0.49 | 0.07 | -6.80 | 0.0000 |
